# Supplementary figures and images for: Size, shape, and direction matters: Matching secondary genital structures in male and female mites using multiple microscopy techniques and 3D modeling
Source: PLoS One. 2021 Aug 18;16(8):e0254974. doi: 10.1371/journal.pone.0254974 (PMC8372888; doi:10.1371/journal.pone.0254974)

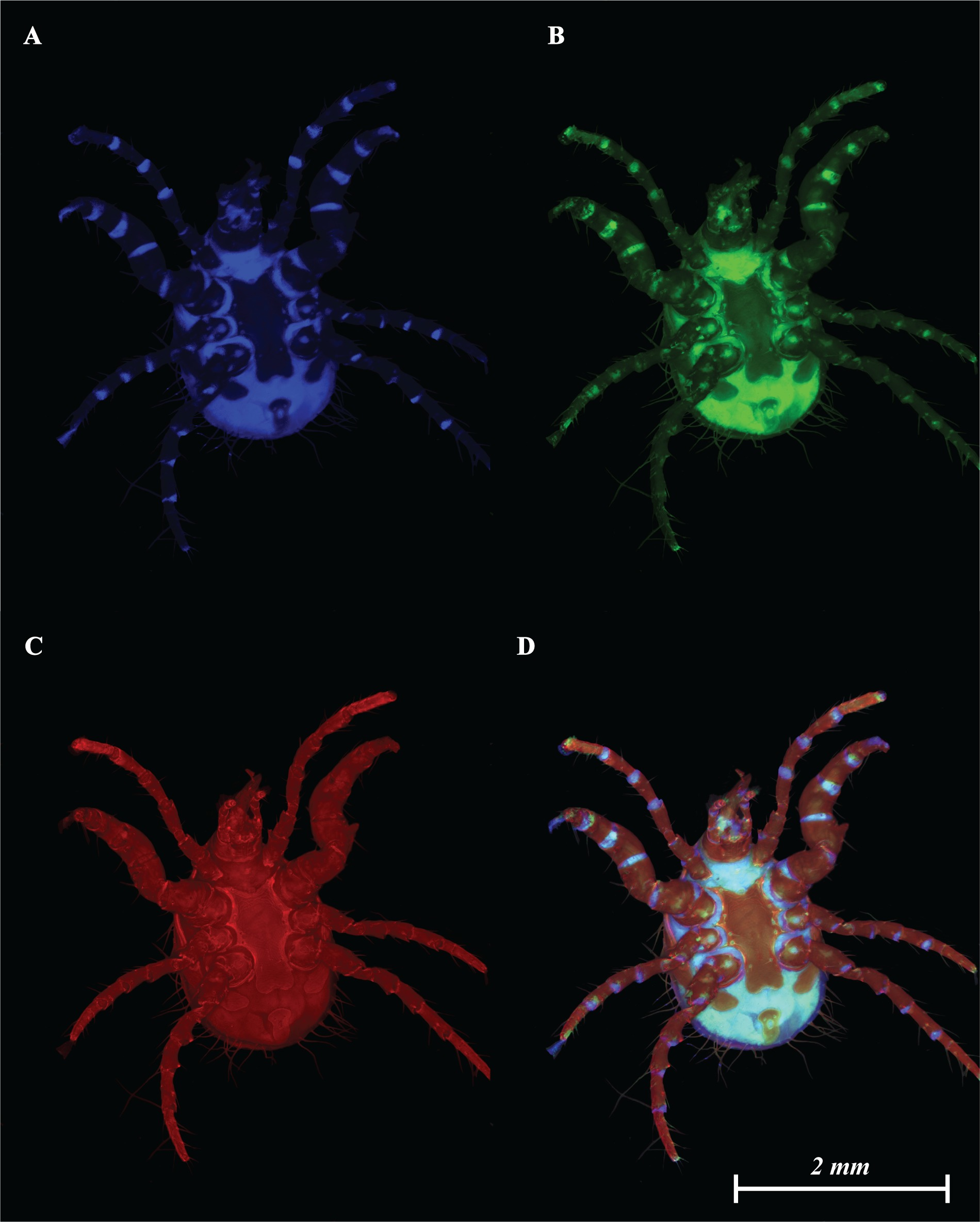

Supplement: S1 Fig — Fluorescence stereomicroscopy image: (A) blue filter (B) green filter (C) red filter (D) combined. (TIF) [file pone.0254974.s001.tif]

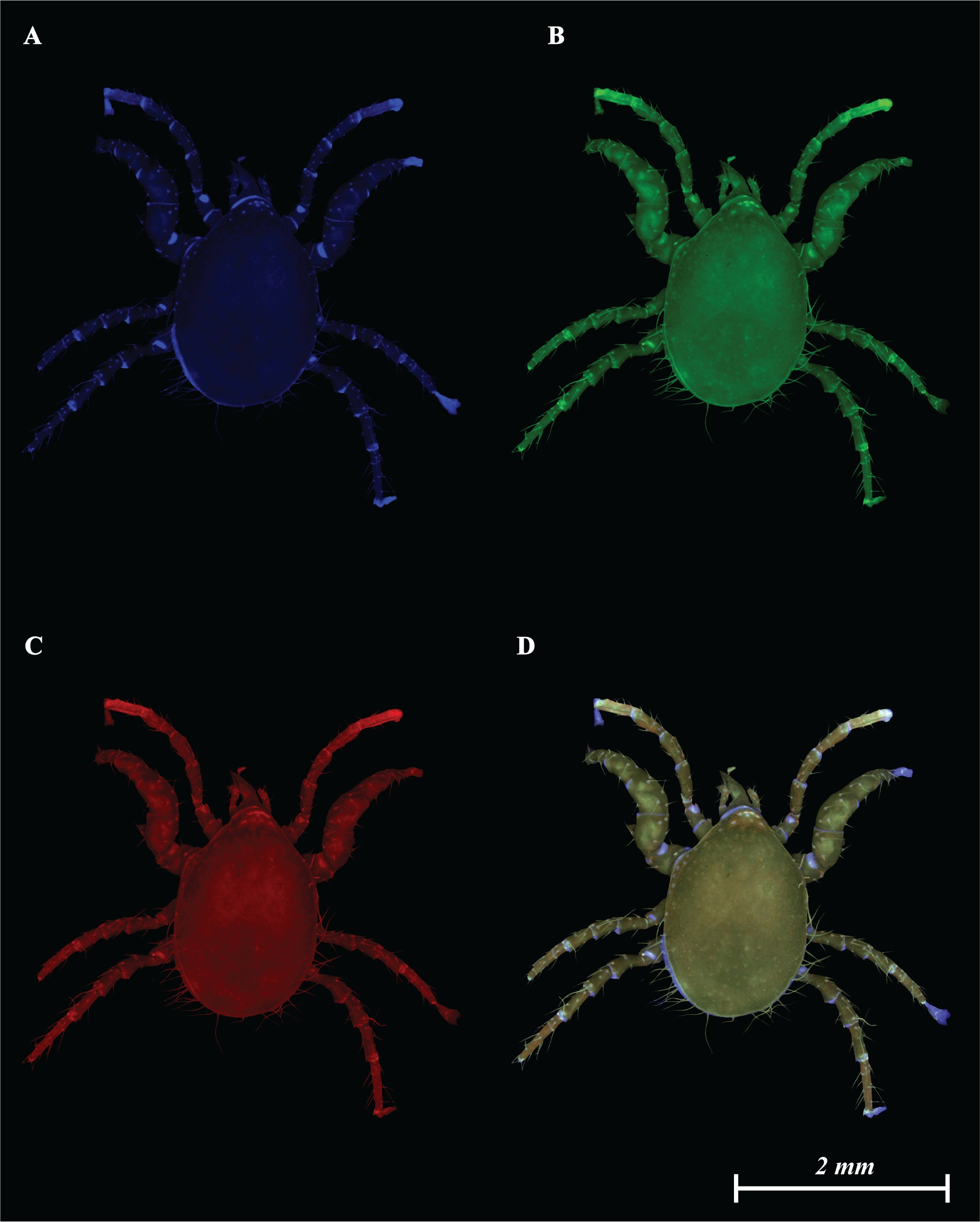

Supplement: S2 Fig — Fluorescence stereomicroscopy image: (A) blue filter (B) green filter (C) red filter (D) combined. (TIF) [file pone.0254974.s002.tif]

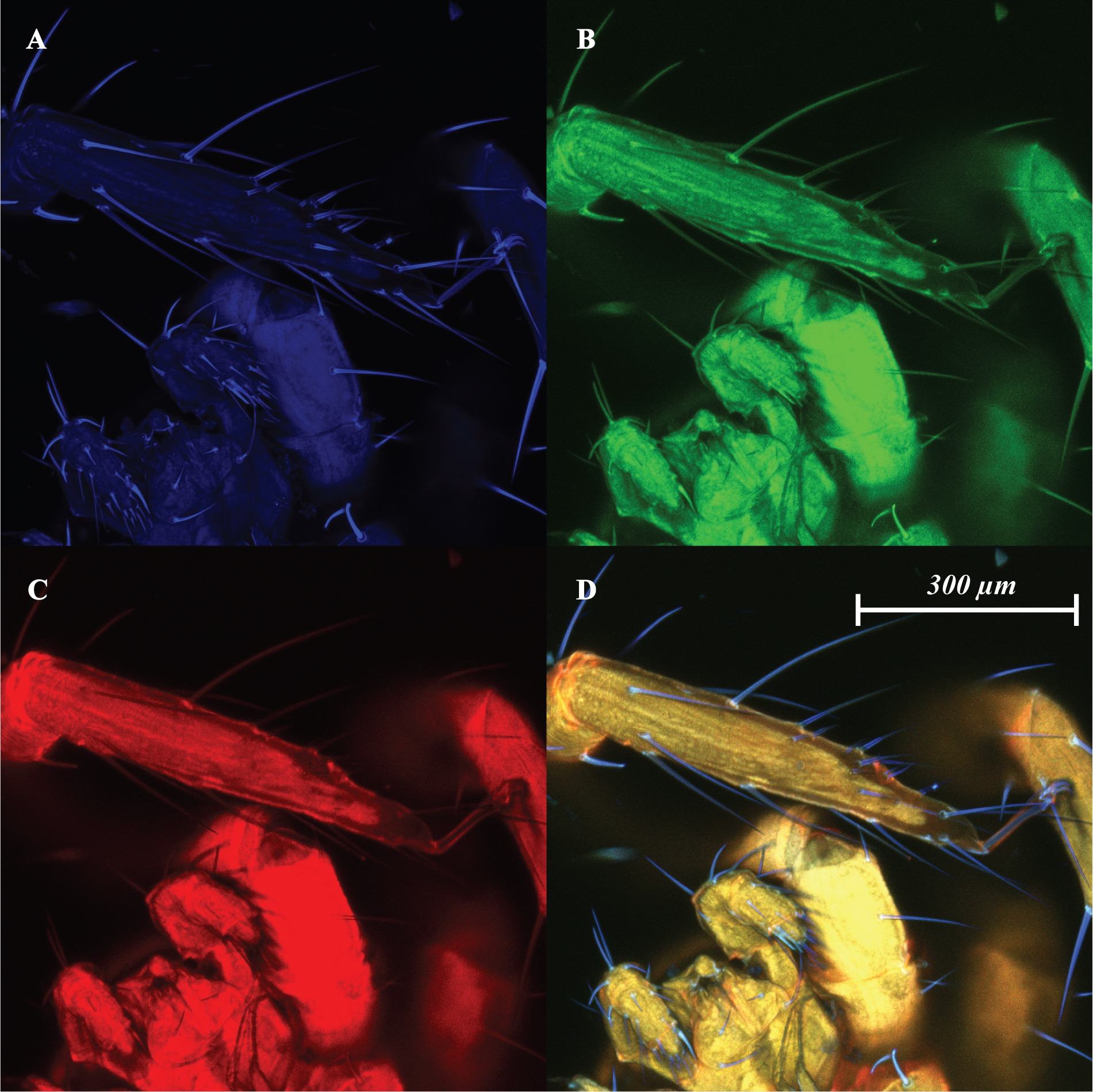

Supplement: S3 Fig — MIP images of tarsus I with CLSM: (A) blue channel (B) green channel (C) red channel (D) combined. (TIF) [file pone.0254974.s003.tif]

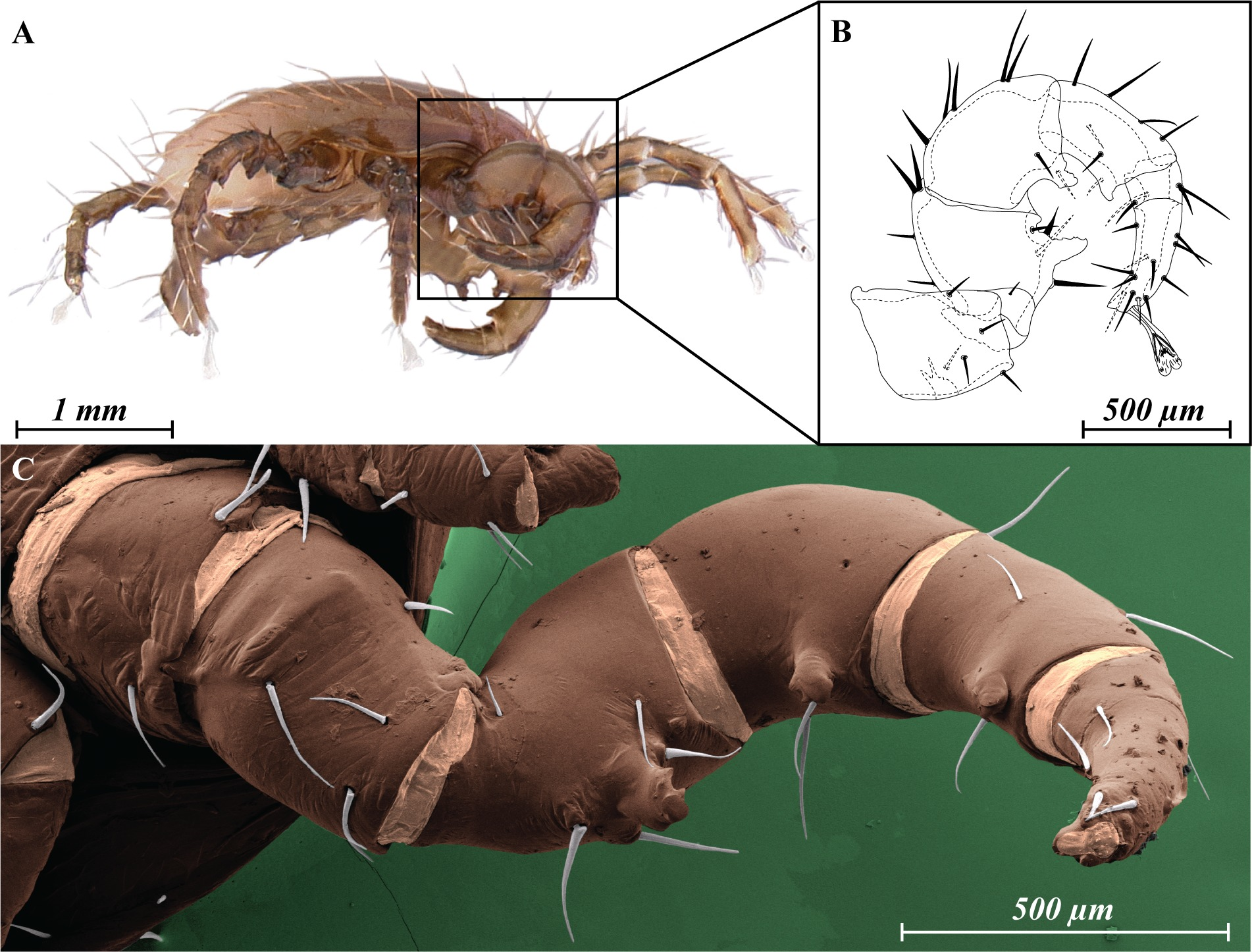

Supplement: S4 Fig — Leg II: (A) colorized LT-SEM of lateral view (B) drawing the detail of leg II (C) colorized LT-SEM image. (TIF) [file pone.0254974.s004.tif]

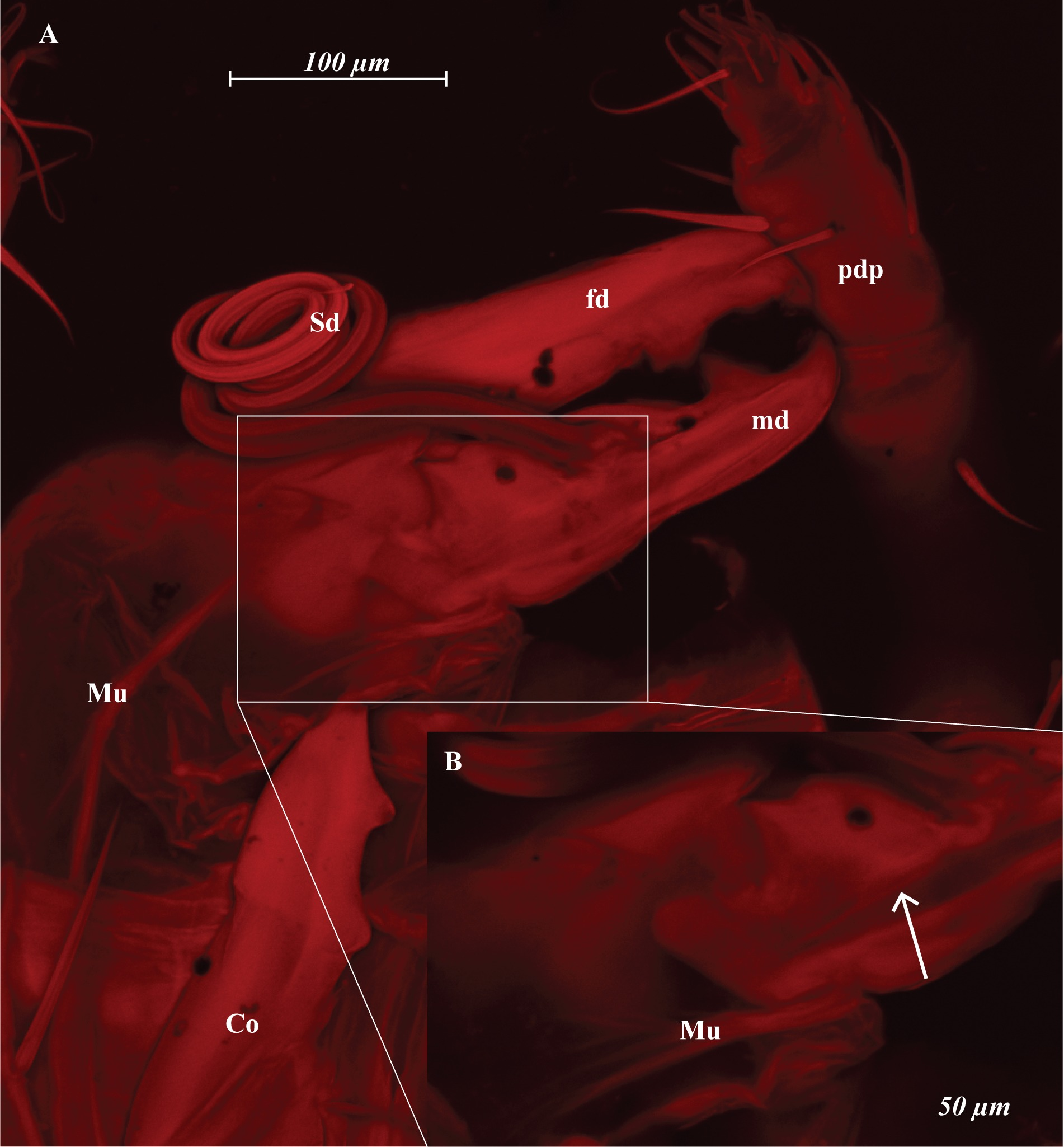

Supplement: S5 Fig — Spermatodactyl and muscle fibers (arrow). Co, corniculi; fd, fixed digit; md, movable digit; Mu, muscle; pdp, pedipalp; Sd, spermatodactyl. (TIF) [file pone.0254974.s005.tif]
