# Supplementary material for: Size, shape, and direction matters: Matching secondary genital structures in male and female mites using multiple microscopy techniques and 3D modeling
Source: PLoS One. 2021 Aug 18;16(8):e0254974. doi: 10.1371/journal.pone.0254974 (PMC8372888; doi:10.1371/journal.pone.0254974)
Supplement: S1 File — 3D rotatable model of female’s sperm access system with coxa III in blue and coxa IV in green. (PDF) [file pone.0254974.s007.pdf]

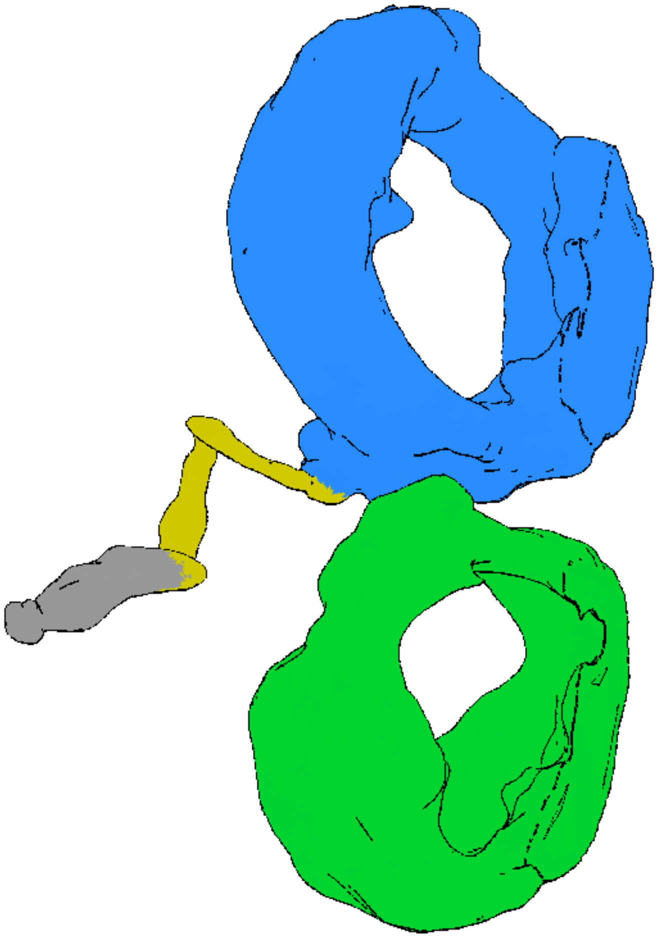

S1 File. ***Megalolaelaps colossus*** **female**. 3D rotatable model of female's sperm access system with coxa III in blue and coxa IV in green.
