# Supplementary material for: Size, shape, and direction matters: Matching secondary genital structures in male and female mites using multiple microscopy techniques and 3D modeling
Source: PLoS One. 2021 Aug 18;16(8):e0254974. doi: 10.1371/journal.pone.0254974 (PMC8372888; doi:10.1371/journal.pone.0254974)
Supplement: S3 File — 3D rotatable model of female’s secondary insemination system with coxa III in blue, major duct in yellow, and calyx in grey. (PDF) [file pone.0254974.s009.pdf]

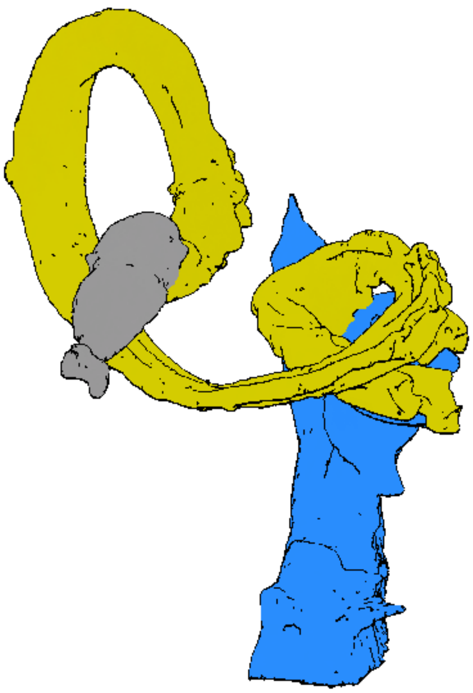

S3 File. ***Megalolaelaps colossus* female**. 3D rotatable model of female's secondary insemination system with coxa III in blue, major duct in yellow, and calyx in grey.
