# Supplementary material for: Size, shape, and direction matters: Matching secondary genital structures in male and female mites using multiple microscopy techniques and 3D modeling
Source: PLoS One. 2021 Aug 18;16(8):e0254974. doi: 10.1371/journal.pone.0254974 (PMC8372888; doi:10.1371/journal.pone.0254974)
Supplement: S1 Table — All figures and 3D models are available at http://morphobank.org/permalink/?P3717. (PDF) [file pone.0254974.s010.pdf]

Figure	Specimen	Instar	Structure	Medium	Facility	Technique	Microscope	Objective	Lasers	Pinhole microns	Excitation Filter	Detection parameters	Size	Z Stack	Voucher number	Morphobank ID
1	*M. colossus*	Male	Chelicerae and spermatodactyl	Hoyer's	CMIF	CLSM	Zeiss LSM880	Plan Apochromat 20x/0.8 M27	561nm DPSS	35	MBS 488/561/633, MBS -405	578 Airyscan	3.5GB	483	OSAL 0099605	M687595
2A	*M. colossus*	Male	Whole body dorsal view	None	ECMU	SEM	TM3030	N/A	N/A	N/A	N/A	N/A	N/A	N/A	N/A	M692293
2B	*M. colossus*	Male	Chelicerae and spermatodactyl	None	ECMU	SEM	TM3030	N/A	N/A	N/A	N/A	N/A	N/A	N/A	N/A	M692293
2C	*M. colossus*	Male	Chelicerae and spermatodactyl	Glycerin	CMIF	CLSM	Olympus FV1000-Filter	UPLFLN 20X NA:0.50	405nm Diode, 488nm Argon, 543nm HeNe1, 633nm HeNe2	120	DM 405/488/543/635	430-470nm for blue, 505-525nm green, 560-660nm yellow-orange, and BA655-755 red	149MB	122	N/A	M692293
2D	*M. colossus*	Male	Chelicerae and spermatodactyl	None	ECMU	LT-SEM	S-4700 LTSEM	N/A	N/A	N/A	N/A	N/A	N/A	N/A	N/A	M692293
2E	*M. colossus*	Male	Chelicerae and spermatodactyl	Hoyer's	CMIF	CLSM	Olympus FV1000-Filter	UPLFLN 40X O NA:1.30	405nm Diode, 488nm Argon, 543nm HeNe1	75	DM 405/488/543	430-470nm blue, 505-525nm green, 560-660nm yellow-orange	663MB	181	N/A	M692293
3A-E	*M. colossus*	Male	Spermatodactyl	Hoyer's	CMIF	CLSM	Olympus FV1000-Filter	UPLFLN 40X O NA:1.30	405nm Diode, 488nm Argon, 543nm HeNe1	75	DM 405/488/543	430-470nm blue, 505-525nm green, 560-660nm yellow-orange	663MB	181	N/A	M692294
4A	*M. colossus*	Female	Dorsal view and internal scheme of coxa II and IV	Glycerin	OSAL	DIC	Nikon Eclipse 90i	Plan Apo 20X/0.5	N/A	N/A	N/A	N/A	N/A	N/A	N/A	M692295
4B	*M. colossus*	Female	Dorsal view and internal scheme of coxa III and IV	N/A	N/A	N/A	Illustrator Drawing based on figure 4D	N/A	N/A	N/A	N/A	N/A	N/A	N/A	N/A	M692295
4C	*M. enceladus*	Female	Coxae III-IV internally	Hoyer's	CMIF	CLSM	Olympus FV1000-Filter	UPLSAPO 20X O NA:0.85	405nm Diode, 488nm Argon, 543nm HeNe1, 633nm HeNe2	80	DM 405/488/543/635	430-470nm blue, 505-525nm green, 560-660nm yellow-orange, and BA655-755 red	284MB	71	NHM120	M692302
4D	*M. colossus*	Female	3D rotatable model of coxa III and IV	Glycerin	ECMU	CLSM	Zeiss LSM710	Fluar 10x/0.50	405nm Diode, 488nm Argon, 561nm DPSS	25	MBS 488/561, MBS 405	410-483nm blue; 495 - 553nm green, 566-703nm red	512MGB	341	N/A	M741030
5	*M. colossus*	Female	Coxae II-IV ventrally	None	ECMU	SEM	TM3030	N/A	N/A	N/A	N/A	N/A	N/A	N/A	N/A	M692296
6A	*M. colossus*	Female	Detail of the spiral organ	Hoyer's	OSAL	DIC	Nikon Eclipse 90i	Plan Apo 40X/0.75	N/A	N/A	N/A	N/A	N/A	N/A	N/A	M692297
6B	*M. colossus*	Female	Detail of the spiral organ	Glycerin	OSAL	DIC	Nikon Eclipse 90i	Plan Apo 40X/0.75	N/A	N/A	N/A	N/A	N/A	N/A	N/A	M692297
6C	*M. colossus*	Female	Detail of the spiral organ	Glycerin	ECMU	CLSM	Zeiss LSM710	Fluar 10x/0.50	405nm Diode, 488nm Argon, 561nm DPSS	25	MBS 488/561, MBS 405	410-483nm blue; 495 - 553nm green, 566-703nm red	2.1GB	354	N/A	M692297
6D	*M. colossus*	Female	Detail of the spiral organ	Hoyer's	CMIF	CLSM	Zeiss LSM880	Plan-Neofluar 40x/1.30 Oil DIC M27	405nm Diode, 561nm DPSS	35	MBS 488/561/633, MBS 405	459/578 Airyscan	6.3GB	503	OSAL 0099606	M692297
7A	*M. colossus*	M, F	Whole body	None	OSUC	St	Leica Z16	1X	N/A	N/A	N/A	N/A	N/A	N/A	N/A	M692315
7B	*M. colossus*	M, F	Whole body lateral view	N/A	ECMU	LT-SEM	S-4700 LTSEM	LTSEM and Illustrator drawing	N/A	N/A	N/A	N/A	N/A	N/A	N/A	M692315
7C	*M. colossus*	Male	3D rotatable model of the chelicerae and spermatodactyl	Hoyer's	CMIF	CLSM	Zeiss LSM880	Plan-Neofluar 40x/1.30 Oil DIC M27	405nm Diode, 561nm DPSS	35	MBS 488/561/633, MBS -405	459/578 Airyscan	6.3GB	503	OSAL 0099606	M741031
7D	*M. colossus*	Female	3D rotatable model of the chelicerae and spermatodactyl	Glycerin	CMIF	CLSM	Zeiss LSM880	Plan-Apochromat 20x/0.8 M27	561nm DPSS	35	MBS 488/561/633, MBS 405	578 Airyscan	3.5GB	483	OSAL 0099605	M741032
8A-E	*M. colossus*	Male	Internal muscles of the chelicera	None	OSUC	St	Leica Z16	1X	N/A	N/A	N/A	N/A	N/A	N/A	N/A	M692300
S1A-D	*M. colossus*	Male	Whole body ventral view	None	ECMU	FSt	Zeiss AxioZoom	1x 0.25NA Plan-NeoFluor	N/A	N/A	N/A	N/A	N/A	N/A	N/A	M692281
S2A-D	*M. colossus*	Male	Whole body dorsal view	None	ECMU	FSt	Zeiss AxioZoom	1x 0.25NA Plan-NeoFluor	N/A	N/A	N/A	N/A	N/A	N/A	N/A	M692282
S3A-D	*M. colossus*	Larva	Tarsus 1	Glycerin	ECMU	CLSM	Zeiss LSM710	Fluar 10x/0.50	405nm Diode, 488nm Argon, 561nm DPSS	35	MBS 488/561, MBS 405	410-483nm blue; 495 - 553nm green, 566-703nm red	246MB	210	N/A	M692288
S4A	*M. colossus*	Male	Whole body lateral view	None	OSUC	St	Leica Z16	1X	N/A	N/A	N/A	N/A	N/A	N/A	N/A	M692298
S4B	*M. colossus*	Male	Leg II	None	OSAL	DIC	Illustration	N/A	N/A	N/A	N/A	N/A	N/A	N/A	N/A	M692298
S4C	*M. colossus*	Male	Leg II ventral view	None	ECMU	LT-SEM	S-4700 LTSEM	N/A	N/A	N/A	N/A	N/A	N/A	N/A	N/A	M692298
S5A-B	*M. enceladus*	Male	Chelicera and spermatodactyl	Hoyer's	ECMU	CLSM	Zeiss LSM710	Fluar 10x/0.50	405nm Diode, 488nm Argon, 561nm DPSS	25	405nm Diode, 488nm Argon,543nm HeNe1, 633nm HeNe2	410-483nm blue; 495 - 553nm green, 566-703nm red	246 MB	82	N/A	M692301

S1 Table. **Individual parameters for the images.** All figures and 3D models are available at http://morphobank.org/permalink/?P3717
