# Supplementary material for: Size, shape, and direction matters: Matching secondary genital structures in male and female mites using multiple microscopy techniques and 3D modeling
Source: PLoS One. 2021 Aug 18;16(8):e0254974. doi: 10.1371/journal.pone.0254974 (PMC8372888; doi:10.1371/journal.pone.0254974)
Supplement: Attachment — Submitted filename: PONE-D-20-28641_reviewer.pdf [file pone.0254974.s011.pdf]

PLOS ONE

Size, shape, and direction matters: recognizing genital coevolution using multi-microscopy technologies and 3D modeling

--Manuscript Draft--

**Manuscript Number:**	PONE-D-20-28641
**Article Type:**	Research Article
**Full Title:**	Size, shape, and direction matters: recognizing genital coevolution using multi-microscopy technologies and 3D modeling
**Short Title:**	Recognizing genital coevolution using multi-microscopy technologies and 3D modeling
**Corresponding Author:**	Orlando Combita-Heredia<br>Ohio State University<br>Columbus, OH UNITED STATES
**Keywords:**	Sexual conflict; Mesostigmata; confocal laser scanning microscopy; low-temperature scanning electron microscopy
**Abstract:**	Studies of female genital structures have generally lagged behind comparable studies of male genitalia, in part because of an assumption of a lower level of variability, but also because internal genitalia is much more difficult to study. Here, we examine these structures in both sexes of a mite Megalolaelaps colossus, with highly modified genital structures in the male. To do so we employed multiple microscopy techniques including video stereomicroscopy, fluorescence microscopy, low-temperature scanning electron microscopy (LT-SEM), and confocal laser scanning microscopy (CLSM). Both LT-SEM and CLSM are well suited for obtaining surface images. However, CLSM also proved to be a valuable technique for observing internal anatomical structures. Female mites have secondary genital structures that match those of the males in complexity. Moreover, the long and coiled spermatodactyl on the chelicera of the males largely matches an internal spiral structure in the females in shape, size, and direction, in accordance with expectations under a genital coevolution model. These microscopy technologies, along with computerized imaging enhancement and 3D modeling techniques, enabled the development of hypotheses for the mechanics of mating and sexual selection.
**Order of Authors:**	Orlando Combita-Heredia
	Connor J Gulbronson
	Ronald Ochoa
	Edwin Javier Quintero-Gutierrez
	Gary Bauchan
	Hans Klompen
**Additional Information:**	
**Question**	**Response**
**Financial Disclosure**<br><br>Enter a financial disclosure statement that describes the sources of funding for the work included in this submission. Review the submission guidelines for detailed requirements. View published research articles from *PLOS ONE* for specific examples.<br><br>This statement is required for submission	(OCH) Doctorados en el Exterior Grant 568-2012- C.C. 80048516 Colciencias, Colombia. https://minciencias.gov.co/quienes_somos/sobre-colciencias

Powered by Editorial Manager® and ProduXion Manager® from Aries Systems Corporation

and **will appear in the published article** if the submission is accepted. Please make sure it is accurate.<br><br>**Unfunded studies**<br>Enter: *The author(s) received no specific funding for this work.*<br><br>**Funded studies**<br>Enter a statement with the following details:<br>• Initials of the authors who received each award<br>• Grant numbers awarded to each author<br>• The full name of each funder<br>• URL of each funder website<br>• Did the sponsors or funders play any role in the study design, data collection and analysis, decision to publish, or preparation of the manuscript?<br>• **NO** - Include this sentence at the end of your statement: *The funders had no role in study design, data collection and analysis, decision to publish, or preparation of the manuscript.*<br>• **YES** - Specify the role(s) played.<br><br>* typeset	
**Competing Interests**<br><br>Use the instructions below to enter a competing interest statement for this submission. On behalf of all authors, disclose any competing interests that could be perceived to bias this work—acknowledging all financial support and any other relevant financial or non-financial competing interests.<br><br>This statement **will appear in the published article** if the submission is accepted. Please make sure it is accurate. View published research articles from *PLOS ONE* for specific examples.	The authors have declared that no competing interests exist.

**NO authors have competing interests**<br><br>Enter: *The authors have declared that no competing interests exist.*<br><br>**Authors with competing interests**<br><br>Enter competing interest details beginning with this statement:<br><br>*I have read the journal's policy and the authors of this manuscript have the following competing interests: [insert competing interests here]*<br><br>* typeset	
**Ethics Statement**<br><br>Enter an ethics statement for this submission. This statement is required if the study involved:<br><br>• Human participants<br>• Human specimens or tissue<br>• Vertebrate animals or cephalopods<br>• Vertebrate embryos or tissues<br>• Field research<br><br>Write "N/A" if the submission does not require an ethics statement.<br><br>General guidance is provided below. Consult the submission guidelines for detailed instructions. **Make sure that all information entered here is included in the Methods section of the manuscript.**	N/A

Powered by Editorial Manager® and ProduXion Manager® from Aries Systems Corporation

**Format for specific study types**<br><br>**Human Subject Research (involving human participants and/or tissue)**<br>• Give the name of the institutional review board or ethics committee that approved the study<br>• Include the approval number and/or a statement indicating approval of this research<br>• Indicate the form of consent obtained (written/oral) or the reason that consent was not obtained (e.g. the data were analyzed anonymously)<br><br>**Animal Research (involving vertebrate animals, embryos or tissues)**<br>• Provide the name of the Institutional Animal Care and Use Committee (IACUC) or other relevant ethics board that reviewed the study protocol, and indicate whether they approved this research or granted a formal waiver of ethical approval<br>• Include an approval number if one was obtained<br>• If the study involved *non-human primates*, add *additional details* about animal welfare and steps taken to ameliorate suffering<br>• If anesthesia, euthanasia, or any kind of animal sacrifice is part of the study, include briefly which substances and/or methods were applied<br><br>**Field Research**<br><br>Include the following details if this study involves the collection of plant, animal, or other materials from a natural setting:<br>• Field permit number<br>• Name of the institution or relevant body that granted permission	
**Data Availability**<br><br>Authors are required to make all data underlying the findings described fully available, without restriction, and from the time of publication. PLOS allows rare exceptions to address legal and ethical concerns. See the PLOS Data Policy and FAQ for detailed information.	Yes - all data are fully available without restriction

A Data Availability Statement describing where the data can be found is required at submission. Your answers to this question constitute the Data Availability Statement and **will be published in the article**, if accepted.<br><br>**Important:** Stating 'data available on request from the author' is not sufficient. If your data are only available upon request, select 'No' for the first question and explain your exceptional situation in the text box.<br><br>Do the authors confirm that all data underlying the findings described in their manuscript are fully available without restriction?	
**Describe where the data may be found in full sentences. If you are copying our sample text, replace any instances of XXX with the appropriate details.**<br><br>• If the data are **held or will be held in a public repository**, include URLs, accession numbers or DOIs. If this information will only be available after acceptance, indicate this by ticking the box below. For example: *All XXX files are available from the XXX database (accession number(s) XXX, XXX.*).<br>• If the data are all contained **within the manuscript and/or Supporting Information files**, enter the following: *All relevant data are within the manuscript and its Supporting Information files.*<br>• If neither of these applies but you are able to provide **details of access elsewhere**, with or without limitations, please do so. For example:<br><br>*Data cannot be shared publicly because of [XXX]. Data are available from the XXX Institutional Data Access / Ethics Committee (contact via XXX) for researchers who meet the criteria for access to confidential data.*<br><br>*The data underlying the results presented in the study are available from (include the name of the third party*	All files figures 1, 2, 3, 4 , 5, 6, 7, 8, 9, S1, S2, S3, S4, S5, and S6 are available from the database: https://morphobank.org/index.php/MyProjects/List/select/project_id/3717. Accession numbers: M687595, M692293, M692294, M692295, M692281, M692282, M692288, M692294, M692294, M692295, M692296, M692297, M692297, M692297, M692297, M692315, M692315, M692311, M692314, M692298, M692299, M692300, M692299, M692300, M692301, M692302

and contact information or URL).<br>• This text is appropriate if the data are owned by a third party and authors do not have permission to share the data.<br><br>* typeset	
Additional data availability information:	Tick here if the URLs/accession numbers/DOIs will be available only after acceptance of the manuscript for publication so that we can ensure their inclusion before publication.

Powered by Editorial Manager® and ProduXion Manager® from Aries Systems Corporation

Manuscript

Click here to access/download;Manuscript;Genital_Coevolution_PLOSBio_co

Size, shape, and direction matters: recognizing genital coevolution using multi-microscopy technologies and 3D modeling

Orlando Cómbita-Heredia[1,2◕], Connor J. Gulbronson[3¤], Ronald Ochoa[4], Edwin Javier Quintero-Gutiérrez[5], Gary Bauchan[6], Hans Klompen[1◕],

1 Acarology Laboratory, Ecology Evolution and Organismal Biology EEOB, Ohio State University, Columbus, Ohio, United States of America
2 Centro de Investigación en Acarología, Bogotá, Colombia
3 Oak Ridge Institute for Science and Education (ORISE) Postdoctoral Fellow. Floral and Nursery Plant Research Unit, United States Department of Agriculture, Agricultural Research Service, U.S. National Arboretum, Beltsville, Maryland, United States of America
4 Agricultural Research Service, Systematic Entomology Laboratory, United States Department of Agriculture, Beltsville, Maryland, United States of America
5 Instituto Colombiano Agropecuario (ICA), Manizales, Colombia
6 Agricultural Research Service, Soybean Genomics and Enhancement Laboratory, Electron and Confocal Microscopy Unit, United States Department of Agriculture, Beltsville, Maryland, United States of America

◕These authors contributed equally to this work.
¤Current Address: Indiana Center for Biological Microscopy, Indiana University, Indianapolis, Indiana, United States of America
* combitaheredia.1@osu.edu

1 Abstract

Studies of female genital structures have generally lagged behind comparable studies of male genitalia, in part because of an assumption of a lower level of variability, but also because internal genitalia is much more difficult to study. Here, we examine these structures in both sexes of a mite, *Megalolaelaps colossus*, with highly modified genital structures in the male. To do so we employed multiple microscopy techniques including video stereomicroscopy, fluorescence microscopy, low-temperature scanning electron microscopy (LT-SEM), and confocal laser scanning microscopy (CLSM). Both LT-SEM and CLSM are well suited for obtaining surface images. However, CLSM also proved to be a valuable technique for observing internal anatomical structures. Female mites have secondary genital structures that match those of the males in complexity. Moreover, the long and coiled spermatodactyl on the chelicera of the males largely matches an internal spiral structure in the females in shape, size, and direction, in accordance with expectations under a genital coevolution model. These microscopy technologies, along with computerized imaging enhancement and 3D modeling techniques, enabled the development of hypotheses for the mechanics of mating and sexual selection.

2 Introduction

Sexual selection may be a major influence on the evolution of genital structures [1]. In this context, the emphasis has been on studies of male genitalia, with considerably fewer

studies focused on female genital structures. This male bias comes in part from the assumption that female genital structures are less variable [2], and thus of less interest, but also for the practical reason that female genitalia are usually internal and harder to analyze. Simultaneous comparative studies of female and male genital structures are even less common [2] [3]. Yet correlation in morphology and/or general compatibility in shape between male and female could be used as an indicator of genital coevolution [4]. Moreover, comparative studies of genitalic morphology can help in determining mechanisms of sexual selection such as sperm competition, cryptic female choice or sexual conflict [3] [5]. Mites (Acari) have not been exploited very often in studies of sexual selection. While some mites show very limited sexual dimorphism and genitalic specialization, others display quite spectacular modifications. Males in the infraorders Dermanyssina and Heterozerconina (Parasitiformes: Mesostigmata) have a specialized structure (the spermatodactyl) on their chelicera which acts as a sperm transfer organ (gonopod). Females of Dermanyssina and Heterozerconina (usually) have a modified insemination system including a pair of secondary genital openings (solenostomes) often located near coxae III or IV [6] [7]. This system of sperm transfer (podospermy) differs from the presumed primitive system (tocospermy) in Mesostigmata where the male uses unmodified chelicera to transfer a sperm packet directly to the primary genital opening (ovipore) of the female [8]. The spermatodactyl is relatively simple in many Dermanyssina, but in some it has become quite complex. For example, males of *Megalolaelaps colossus* Cómbita-Heredia and Quintero-Gutiérrez (Dermanyssina: Megalolaelapidae) present a strikingly complex spermatodactyl (Figure 1) [9]. As in all Dermanyssina, the spermatodactyl in *M. colossus* is located on the movable digit of the chelicera. It is highly coiled, resembling a corkscrew that if uncoiled could be half as long as the body (Figure 2a-c). Knowledge of mesostigmatid genital systems is uneven. Several studies on internal reproductive structures of podospermic female mesostigmatid mites have led to the distinction of two general systems, the laelapid and phytoseiid types [7], [10], [11], [12], [13]. Other studies have led to a better understanding of the configuration and development of the spermatodactyl in males [14], [15], [16], [17]. However, males and females have rarely been studied simultaneously with the explicit goal of examining potential coevolution. One possible exception is a study on *Veigaia paradoxa* Willmann, where the authors note that the spermatodactyl is as long as the internal "spiral organ" in the female, supporting Willmann's hypothesis that the spermatodactyl may be inserted in this "spiral organs" [18]. We note that from an evolutionary point of view, secondary structures like spermatodactyl, secondary insemination systems, and associated structures should be included under the category of genital traits, because they are directly involved in copulation [19]. One significant technical challenge in examining the interaction of male and female genital structures is that female reproductive structures are internal, and thus not amenable for study using Scanning Electron Microscopy (SEM). The often clearly 3-dimensional reproductive structures are also difficult to study using standard light microscopy. Transmission Electron Microscopy (TEM) has been used to great effect in previous studies and produces highly detailed images, but this type of analysis is very time consuming and destructive. Confocal Laser Scanning Microscopy (CLSM) is a technique that cannot match the resolution of TEM, but it is fast, non-destructive, can be applied to fluid and slide preserved specimens, and can help reveal 3-dimensional internal and external structures. The goals of this paper are to use multiple microscopy technologies to investigate the following hypotheses: 1) the female secondary genital structures are as complex as the male structures, and 2) the secondary insemination system in females of *M. colossus* matches the complexity of the male spermatodactyl, as expected under a hypothesis of genital coevolution.

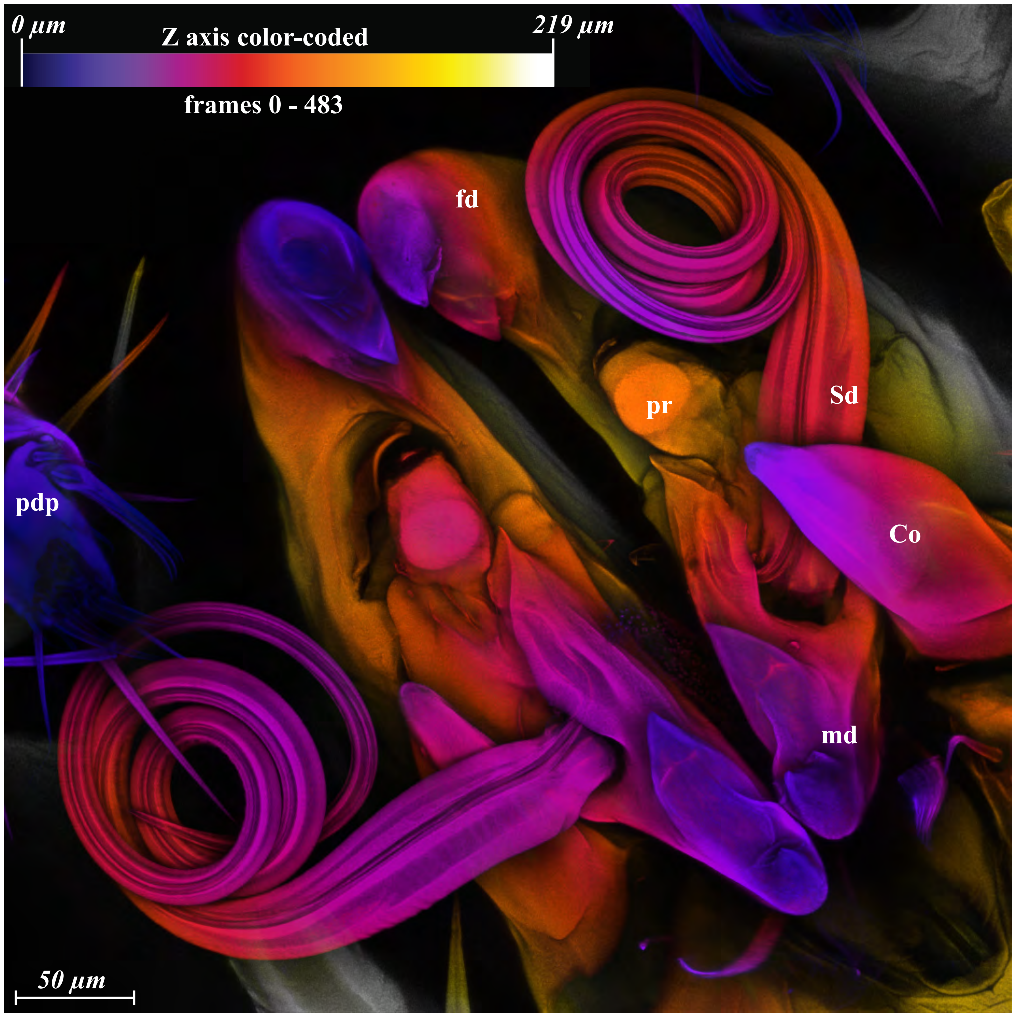

Fig 1. *Megalolaelaps colossus* male, maximum intensity projection (MIP) of CLSM image of the spermatodactyl with color coded z axis. Co, corniculi; fd, fixed digit; md, movable digit; pdp, pedipalp; pr, putative reservoir; Sd, spermatodactyl.

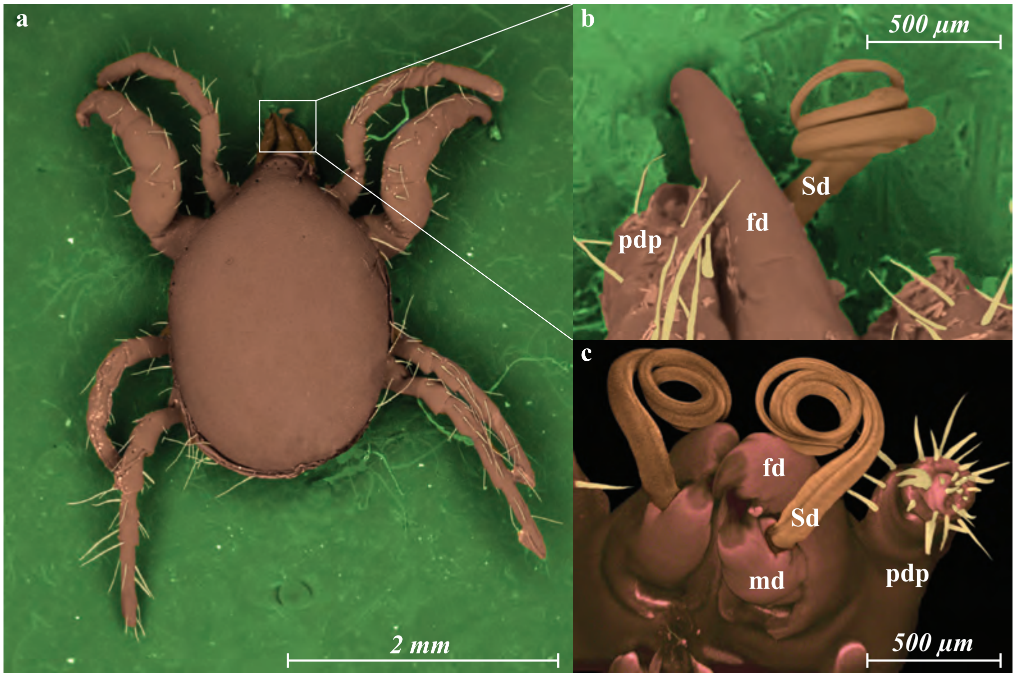

Fig 2. *Megalolaelaps colossus* male (a) dorsal view LT-SEM colorized image (b) detail of the spermatodactyl (c) frontal view of spermatodactyl CLSM colored image. fd, fixed digit; md, movable digit; pdp, pedipalp; Sd, spermatodactyl.

3 Results

LT-SEM in Mesostigmata

LT-SEM is a of great importance because it requires minimal specimen preparation and enables specimens with high water content to be frozen in liquid nitrogen and placed in the vacuum of the SEM and observed in a near natural state without the use of chemical fixatives. Cryo-preparation of mites in combination with the use of high-resolution field emission SEMs has enabled scientists to observe ultra-delicate external structures that have not been previously observable [20]. Black and white SEM images can be colorized to resemble the natural color of the organism or colors can be added to emphasize critical morphological features [21]. Although the LT-SEM allows for detailed studies of the external structures of mites it is not useful for studies of internal structures.

CLSM in Mesostigmata

The steps and variables to be considered when using CLSM for internal and external structures in Mesostigmata are as follows. Collecting methods can follow standard recommendations and protocols (e.g. [6]) because this will not affect the result of the final scan. However, preservation and clearing methods may have a significant impact. For instance, clearing specimens with lactic acid or KOH may cause degradation of internal organs and soft cuticle. The cuticle of mites tends to have a relatively strong level of autofluorescence compared to other arthropods, possibly because of elevated levels of components such as pteridines and resilin [22]. Therefore, use of targeting fluorochromes is only necessary for unsclerotized structures such as muscle or nervous tissue. Autofluorescence signal strength tends to diminish over time and also with exposure to fluorescent light, an effect known as photo bleaching [22]. However,

Mesostigmata preserved in a slide mounting medium such as Hoyer's seem to retain a good amount of autofluorescence and make good quality images. For instance, we photographed one female of *M. enceladus* Berlese using a slide prepared in 1930 with Hoyer's medium (Figure S6). Additionally, photo bleaching does not appear to be a major problem with Mesostigmatid mites, as some of the specimens were scanned more than ten times and the image quality did not degrade. Before starting a CLSM session, a fluorescence stereomicroscopy image was acquired in order to check which filters are most suitable for each specimen (ventral view Figures S1 a–d; S2 a-d; dorsal view). However, these techniques continuously expose the mites to high intensities of UV light which could accelerate photo bleaching. The CLSM test studies showed that the red channel provided the strongest signal in Mesostigmata making it the most suitable for the study of especially hard sclerotized structures. However, the blue and green channels provide added information on setae and some soft cuticle structures like the empodium on the pretarsus (Figures S3 a–d).

Male and Female Reproductive Structures

Males: Previous studies of the gnathosomal structure of *Megalolaelaps* Gorirossi-Bourdeau, mention the spermatodactyl in males but provides few details [23]. The spermatodactyl is relatively long (ca. 1200 μ extended (N=11; 1112 – 1285)). It appears to be movable; it is highly coiled resembling a corkscrew. The left spermatodactyl is directed counterclockwise and the right is oriented clockwise from proximal to distal end. It is located antiaxial on the movable digit. Based on orthogonal views of the 3D images the spermatodactyl seems to be an evagination of the procuticule of the movable digit of the chelicera. The joint between the spermatodactyl and the movable digit (Figures 3a, b) appears to be flexible with an expansion or "head" at the base of the spermatodactyl inserted in a big concave area on the movable digit resembling a synovial saddle joint in vertebrates. Presumably this allows considerable twisting of the spermatodactyl relative to the movable digit. The joint is covered by a thin membrane that's visible under SEM (Figure 3a arrow) but less conspicuous with CLSM (Figure 3b arrow). The entire coiled spermatodactyl fits completely in the gnathosomal cavity or camerostome when the chelicerae are retracted. The sperm delivery system in *M. colossus* starts in the movable digit proper as a duct that connects to a proximal process or "putative reservoir" [17]. After leaving the movable digit and extending into the spermatodactyl, the duct becomes an external groove (Figure 4 a arrows). This system differs from the spermatodactyl in other podospermic species which have an internal sperm duct rather than an external groove. The spermatodactyl in *Megalolaelaps* contains two internal cavities that appear connected to the movable digit and run on either side of the external groove). The cuticle of the spermatodactyl presents a series of coiled rugosities along the internal side (Figure 4b,c arrow) resembling the taenidia of trachea or perhaps more appropriately the annuli-like structures present on the cheliceral shaft of some Uropodina (Mesostigmata), e.g. *Uroactinia* [24]. We assume that these structures permit shape modification, specifically uncoiling, of the spermatodactyl. Additionally, we observed inside each cavity a structure we could not identify that could be unfolded (Figure 4d) or folded (Figure 4e) Females. The secondary genital openings are located on coxae III (Figure 5 arrows). Internally, they open into a cavity in the coxa that may function as a sperm pocket. Subsequently, and in the body proper, they connect to a pair of well sclerotized spiral structures similar to the "spiral organs" in *Veigaia* [11], [25]. The left internal spiral structure has a clockwise direction and the right has a counterclockwise direction starting from the ventral pore to deep within the body. The total average length from the pore to the sclerotized spiral part is 1285 microns (N=9; 1165 – 1205). Based on orthogonal views of the 3D images, the sperm reception system seems to be and

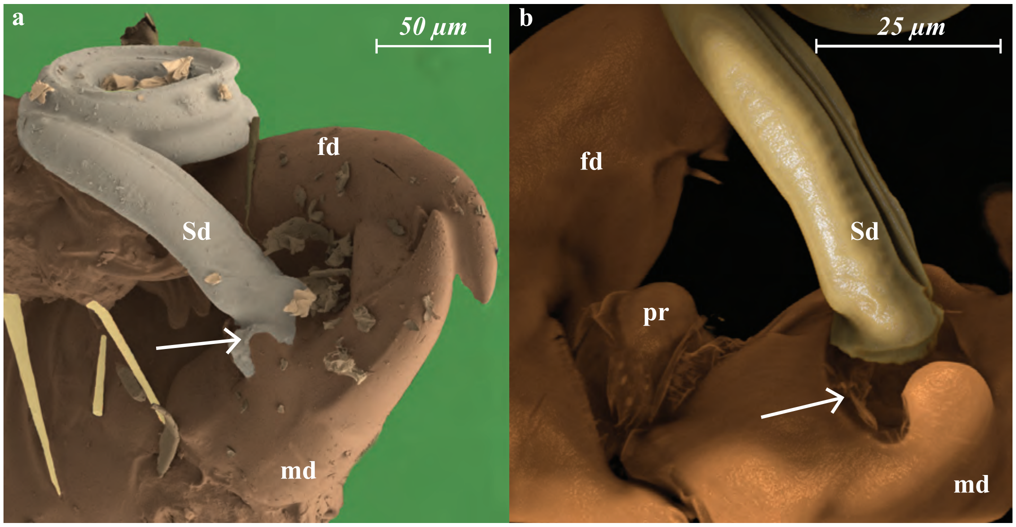

Fig 3. *Megalolaelaps colossus* spermatodactyl male. (a) colorized LT-SEM (b) 3D model based on CLSM. Arrows: articulation between spermatodactyl and movable digit image. fd, fixed digit; md, movable digit; pr, putative reservoir; Sd, spermatodactyl.

invagination of the epicuticle of the coxae. Internally, these spiral structures connect to an elaborate internal secondary insemination system closely resembling homologous structures in other Dermanyssina. Each of the sclerotized structures continues into an unsclerotized structure resembling the sacculus vestibulus in the phytoseiid sperm induction type [6]. Then the sacculus vestibulus-like structure connects to a major duct, which connects to an atrium and a calyx (Figure 6a–d arrow) and finally connects to an inconspicuous vesicle that occupies a large part of the abdomen. Additional minor duct(s) were not observed.

4 Discussion

CLSM in Mesostigmata

This is the first study using CLSM for the study of morphology in Mesostigmata. Our initial assumption, based on previous studies done in mites of the suborder Acariformes [22], [26], [27], [28], [29], [30], [31], [32], [33], was that CLMS would be a successful technique for external and internal morphology, even though mesostigmatid mites have a thicker and more sclerotized cuticle. Although specific parameters needed to be adjusted (see Table 6), we demonstrate that CLSM is indeed a quite useful method to study internal and external anatomy in Mesostigmata. CLSM allowed 3-dimensional reconstruction of the female and male secondary genitalic structures. Confirming the first hypothesis and recent reviews on the topic (e.g. [3]), the female secondary genitalic structures in *M. colossus* are highly complex, including various pockets and a pair of large, sclerotized, spiral structures connecting with the secondary genital openings near coxae III. It is important to note that these structures are in addition to the "standard" unsclerotized ducts of the dermanyssine secondary genitalia.

Matching and Function

The data in this study also confirm the second hypothesis that the female internal genital structures match the male external structures, in accordance with expectations

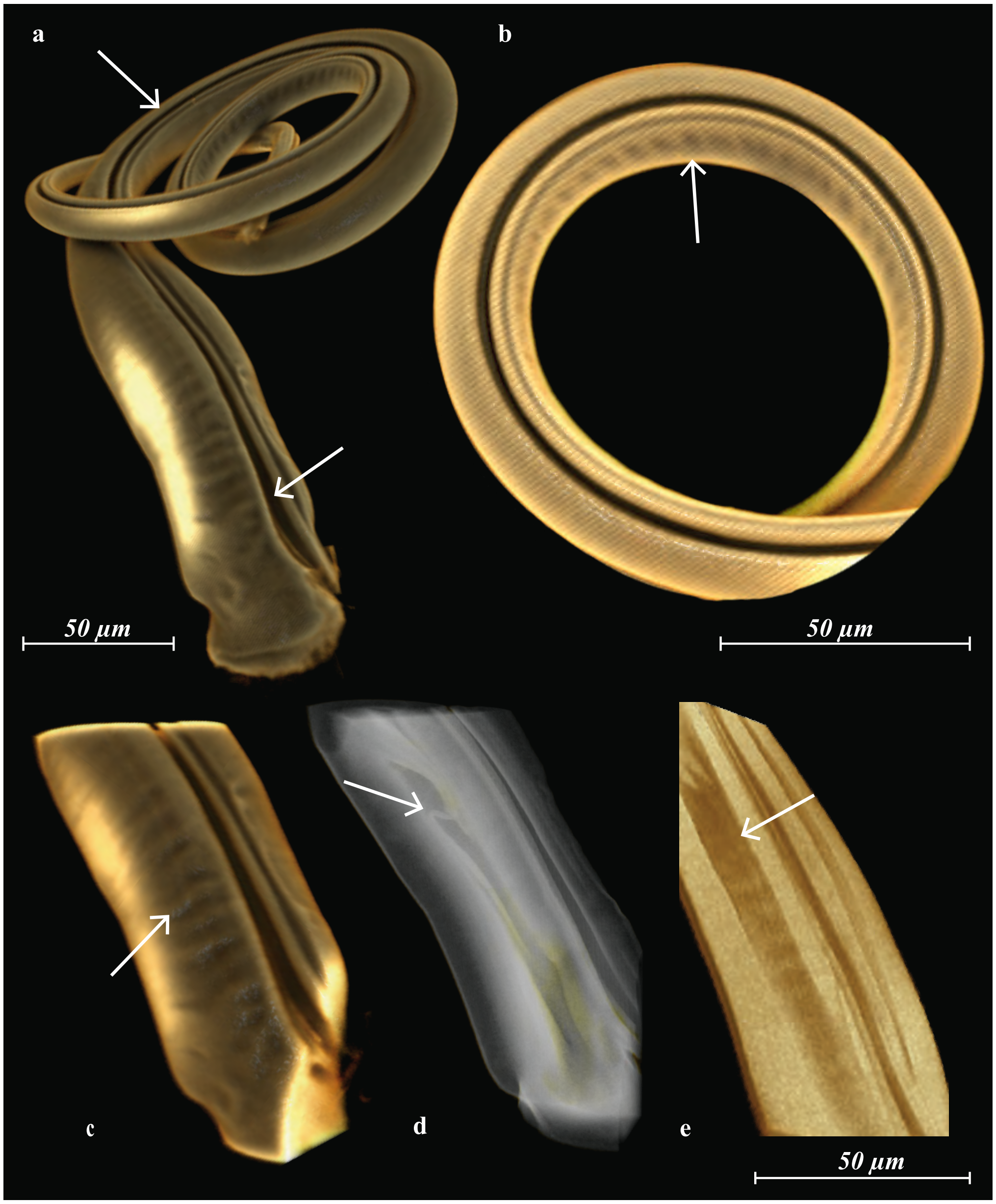

Fig 4. *Megalolaelaps colossus* male spermatodactyl 3D model from CLSM. (a) detail of the external groove (arrows) (b) detail of the distal annuli-like structures (arrow) (c) detail of the proximal annuli-like structures (arrow) (d) detail of the unidentified structure unfolded (arrow) (e) detail of the unidentified structure folded (arrow).

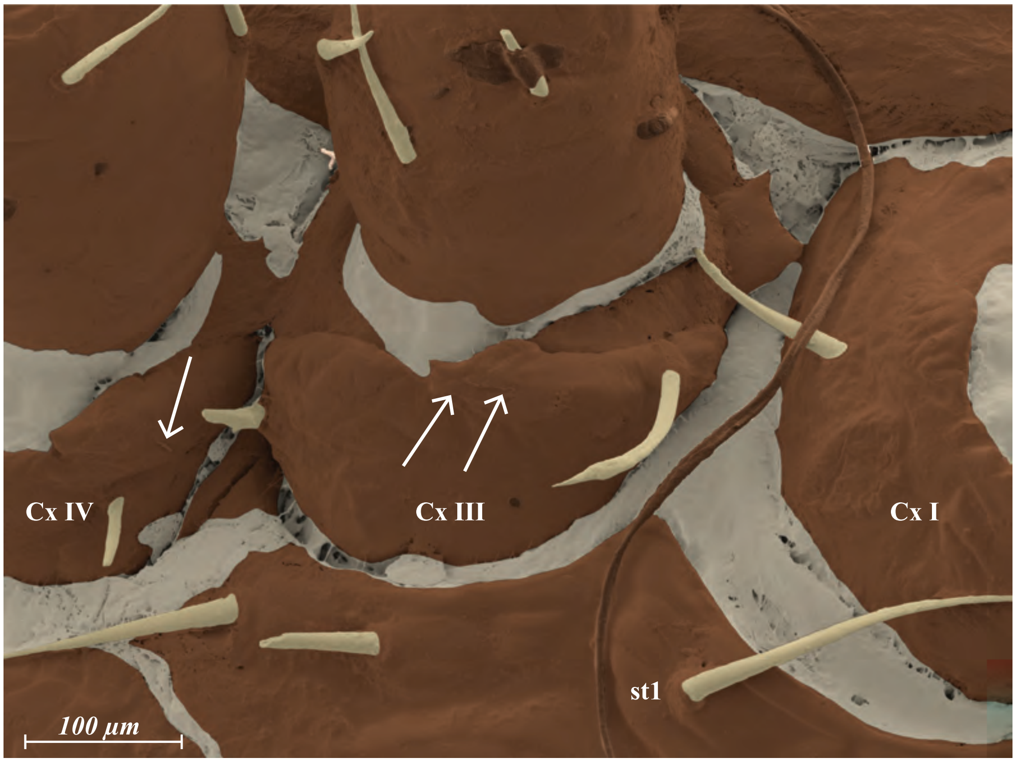

Fig 5. *Megalolaelaps colossus* female ventral LT-SEM colorized image of coxae III – IV. Cx I – III, coxa I – III; st1, sternal setae 1. Possible solenostomes (arrows).

under a genital coevolution scenario. The female internal spiral structure has the same direction and shape as the male spermatodactyl in the venter-to-venter mating position adopted by *M. colossus* (Figure 7). In addition, the total length of the spermatodactyl is similar to the length of the sclerotized part of the female sperm induction system (averages of 1180 μm and 1115 μm, respectively). These observations are consistent with a hypothesis of genital coevolution. The mechanism leading to genital coevolution in this species is less clear. The following options have been considered in various studies of both invertebrates [2], [3] and vertebrates [5]: Matching of morphologically intricate genitalic systems has been studied extensively on invertebrates specially as a type of cryptic female choice known as the lock-and-key mechanism [2], [3]. However, the system here studied resembles some of the genitalic matching studies in vertebrates [5], [19], [34]. For example, the long and coiled vaginal "elaborations" in ducks correlate with the morphology of the phallus. That system has been hypothesized as resulting from sexual selection, specifically sexual conflict [5]. The coiled genitalia in ducks contemplated several alternative explanations for the observed matching pattern of genitalia, specifically natural selection, genitalic homology, and different sexual selection mechanisms such as male competition, female choice and sexual conflict [5]. Here, we considered the same alternatives for genitalic matching by giving some speculative interpretations with the data available on the different scenarios. First, natural selection could explain the development of complex male and female genitalia if the relevant traits were subject to independent selective pressures that could lead to similar morphologies. For instance, the spermatodactyl could have evolved as a feeding accessory (unlikely given that the big coiled spermatodactyl may actually interfere with feeding). For the female, the internal long spirals of the secondary insemination system, could have evolved as a mechanism to avoid contamination during copula since these

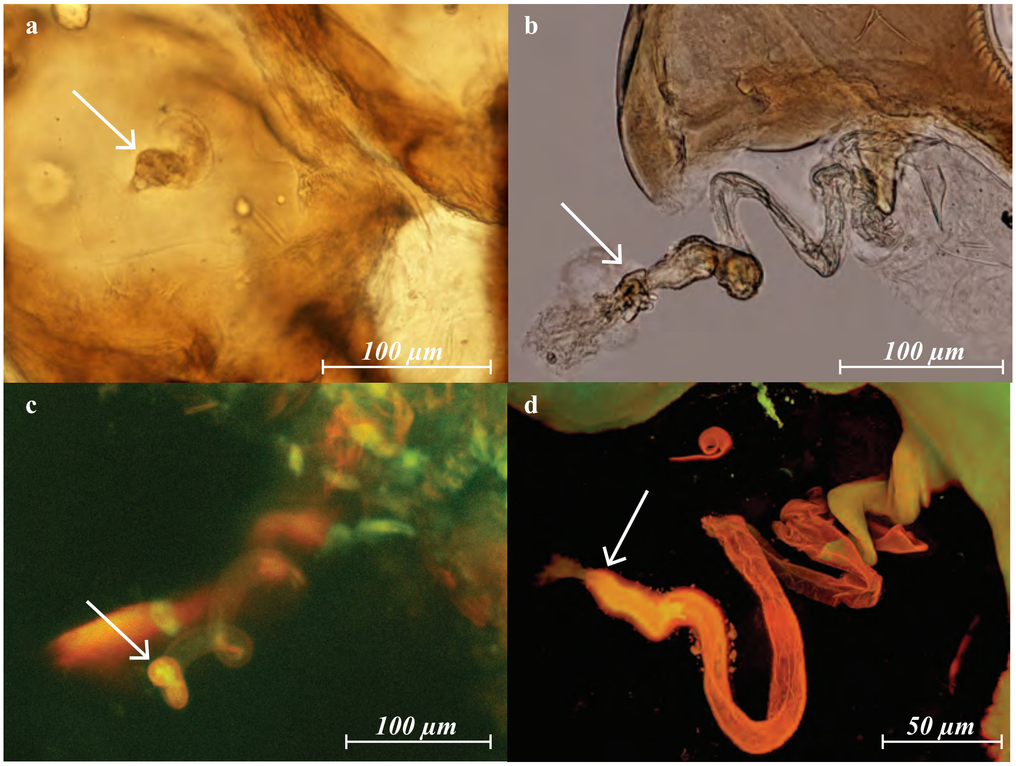

Fig 6. *Megalolaelaps colossus* female secondary insemination system. (a) DIC Image dorsal of cleared specimen (b) DIC image of a dissected specimen (c) MIP images from CLSM of cleared specimen (d) MIP image from CLSM of a dissected specimen.

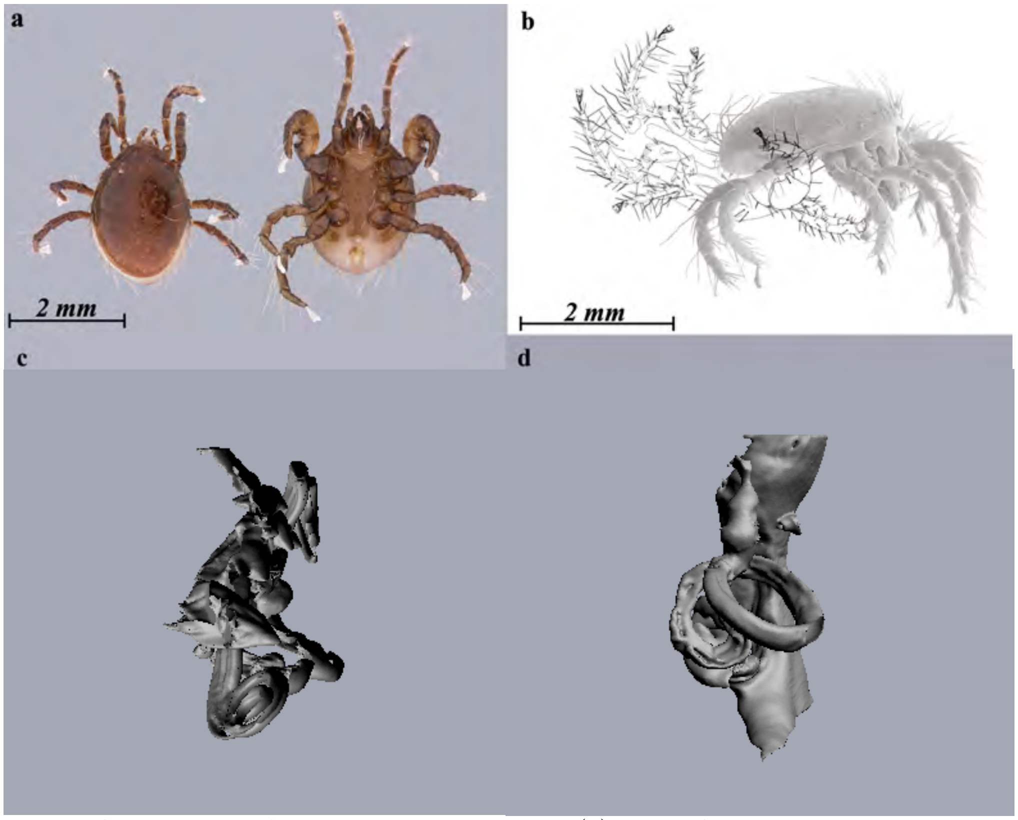

Fig 7. Copula model for *Megalolaelaps colossus* (a) dorsal female and ventral male stereomicroscope images (b) female and male lateral (c) 3D model of spermatodactyl (d) 3D Model of secondary insemination system.

mites live and reproduce in manure. However, this scenario alone does not explain why male and female structures mirror each other in shape, direction and size. Second, homology could explain the extreme and matching morphologies if the affected organs in both sexes were indeed homologous. However, while the primary reproductive elements (e.g. testis, ovary) are homologous for males and females, the spermatodactyl and sperm induction system have different locations and origins (procuticle of the chelicera and epicuticle of the coxae, respectively). Among various mechanisms related to sexual selection a lock-and-key model would seem to be an excellent explanation for the observed matching. The idea is that close matching of female and male genitalia would evolve to avoid inefficient matings with heterospecific partners. The hypothesis was considered unlikely when female genitalia were assumed to be relatively invariant and simple, but with more recent evidence that this assumption is incorrect, the hypothesis has been gaining more support [2], [3]. However, this hypothesis does have some strong predictions, including an expectation of reproductive character displacement in areas of sympatry of multiple closely related species. Most of our observations are based on specimens of M. colossus from Quindio, where only *M. colossus* occurs. However, we (OCH, HK) also collected *M. colossus* in the Amazon region, where it co-occurs with a new species *M.* n. sp. 2. At that site, *M. colossus* has a longer spermatodactyl than *M.* n. sp. 2. (960 μm vs. 773 μm). The lock-and-key hypothesis predicts that this difference would be amplified in a region of species overlap but the reverse was observed: *M. colossus* males from the Amazon region have smaller spermatodactyls than males from Quindio. This makes a lock-and-key mechanism a less likely explanation for the observed genital coevolution. One caveat is that we only had a few males of *M. colossus* from the Amazon region and were not able to assess within population variability. Finally, two alternative mechanisms of sexual selection, as male competition and cryptic female choice, are also not supported by current data and observations. If the exaggerated genitalia were the result of male competition, one would expect other exaggerated morphological characters in males to support that function. However, the only obvious candidate character, the strong legs II with distinct spurs in male *M. colossus* commonly occur in Mesostigmata to hold onto the female during (perhaps forced) mating (Figure S4) and not for male – male interactions. Second, male-male competition and cryptic female choice scenarios are most often invoked in situations with multiple males attempting mating with all available females, and not for species with distinct pair bonding. *Megalolaelaps colossus* are near permanent associates of their beetle hosts, living singly or in pairs on a given beetle. Reproduction is centered on the gallery dug by each beetle pair for its reproduction [35]. In this environment, mating options for the mites appear to be limited to one or maybe two partners, a situation that does not fit these scenarios very well. This leaves as the least unlikely hypothesis sexual conflict [36]. Sexual conflict has been invoked as a major force driving the antagonistic coevolution of physiological, behavioral and/or morphological traits for reproduction [37] and assumes selection in both partners for features that may lead to reproductive dominance over the other, leading to an evolutionary arms race. Under this model if a male has a complex reproductive morphology, female genitalia are assumed to coevolve in complexity to recover control over reproduction. Practically, that would imply that female reproductive structures should show equivalent complexity to that shown in their male counterpart (e.g. size, shape, direction, motion, location among others). One major issue is the question whether there is any conflict between male and female in this species, and if there is, how that would be expressed. Rearing of the mites [35] has solved multiple questions, but not (yet) that one. A final note on the possibility of sexual conflict in this system concerns the pair of secondary pores on coxae IV. These pores have a similar configuration as the pores on coxae III, but they lead to dead-end sperm pocket-like structures and well sclerotized (but smaller) spirals

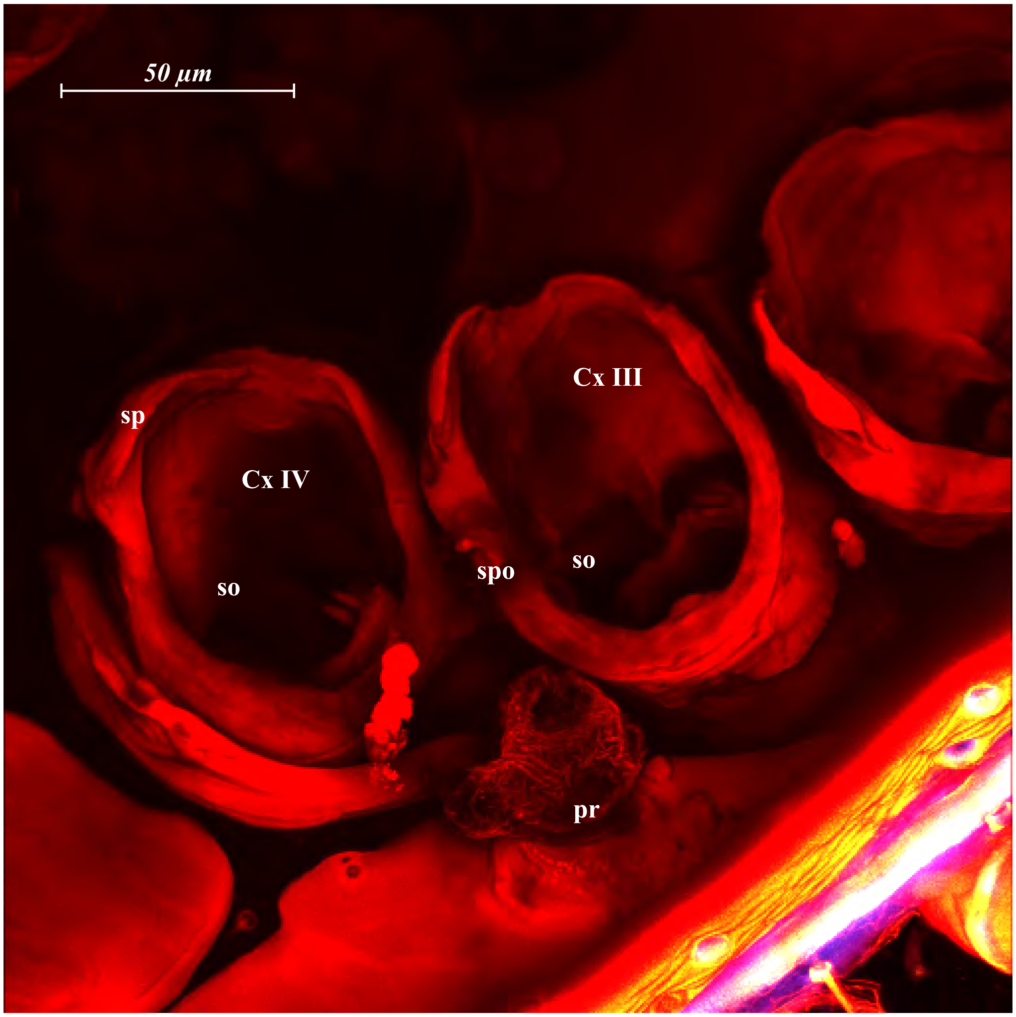

Fig 8. *Megalolaelaps colossus* female internal view of coxa II and IV. so, solenostome; spo, spiralorgan; Cx III – IV, coxa III – IV; pr, peritreme; sp, sperm pocket.

(Figure). These structures might result from serial duplication/homology without any direct significance, but they would also fit into a sexual conflict model as deflection options for females trying to avoid mating with non-preferred males. Our observations raise a second question, dealing with the mechanics of mating in *M. colossus*. While the match of male and female genital structures suggests extensive introduction of the spermatodactyl into the female secondary insemination system, how is this done given the strong sclerotization of both male and female systems? In fact, there are two, somewhat, separate questions: 1) How can the tightly wound structure of the male spermatodactyl be introduced in the spiral of the female structures, and 2) what can be the mechanism to power that introduction? The answer to the first question is relatively straightforward. The articulation of the spermatodactyl with the movable digit, as well as the ring- or annulus-like structures observed in the spermatodactyl proper should allow considerable flexibility of the spermatodactyl during insertion into the long coiled female secondary insemination system. Laboratory experiments with the spermatodactyl demonstrated that the articulation is movable and also that the spermatodactyl can indeed unfold from its coiled resting state (Figure 9). The same experiment (Video S1) showed that the spermatodactyl resumed its coiled shape after

release, suggesting that coiling results from elasticity. As for the second question, this is much less clear. Insertion by introducing the tip of the spermatodactyl and continuous pressure through the male chelicera seems unlikely, as it is unclear how such pressure could uncoil the spermatodactyl. That would suggest some pressure from within the male chelicera to effect uncoiling. One option would be musculature in, or at the base of, the spermatodactyl. Potential muscle fiber-like structures were observed in *M. enceladus* under CLSM (S5 arrow) but presence of such fibers could not be confirmed for *M. colossus*. It is also unclear what the origin of such muscles, if present, would be. An alternative would be hydrostatic pressure, perhaps created in the movable or fixed digit and expressed through the cavities in the spermatodactyl. Unfortunately, available evidence is insufficient to test such a hypothesis. In summary, *Megalolaelaps colossus* has highly complex secondary genital structures in both sexes, with structures in females and males that are largely matching. To resolve the mechanism for this case of genitalic coevolution will require substantial additional data, but with the advent of new microscopic techniques we have the ability to assemble the basic data to start asking such questions.

5 Materials and Methods

Collecting Data

Mites were found associated with dung beetles, *Oxysternon conspicillatum* (Weber) in two localities. A mixed crop of coffee and plantains located in “El Bosque” farm, Calarcá, Quindío, Colombia, 4°31’09.7”N, 75°37’35.9”W, and Esteban Carillo’s farm, Km 11 Tarapacá road, Leticia, Amazonas, Colombia (4°05’44.7” S 69°57’00.9” W). Beetles were collected using non-lethal pitfall traps. Mites were removed under a dissecting scope and preserved in 95 percent ethanol.

Light Stereomicroscopy (St)

Images were obtained using a Leica Z16 APOA stereomicroscope (Buffalo Grove, IL) with a 1X objective lens and equipped with a JVC KY-F75U digital camera (Hachioji, Japan). Stacked images were merged and processed using Combine ZP (https://combinezp.software.informer.com/) and AutoMontage Pro (http://www.syncroscopy.com/) at the C.A. Triplehorn Insect Collection (OSUC) of the Ohio State University (OSU)

Fluorescence Stereomicroscopy (FSt)

Images of the mites were obtained by placing them in a petri-dish with 70 percent ethanol. A Zeiss AxioZoom microscopy system (Thornwood, NY) at the Electron and Confocal Microscopy Unit (ECMU) at the USDA-ARS in Beltsville, Maryland. The images were obtained using a 1x 0.25NA PlanNeoFluor objective. Fluorescence microscopy was accomplished using a 200 watt mercury vapor lamp (HXP Short Arc Lamp, Thornwood, NY) with a filter set for DAPI with excitation at 335–383nm, beam splitter 395nm and emission at 420–470m; GFP with excitation at 450–490nm, beam splitter 495nm and emission at 500–550nm; mRFP with excitation at 559–585nm, beam splitter 590nm and emission at 600–690nm. Fluorescence was captured using an AxioCam 506 mono camera. Zen 2 Pro Blue (Thornwood, NY) 64-bit software was used to capture 15–20 Z-stack images using extended depth of focus to produce 2D images.

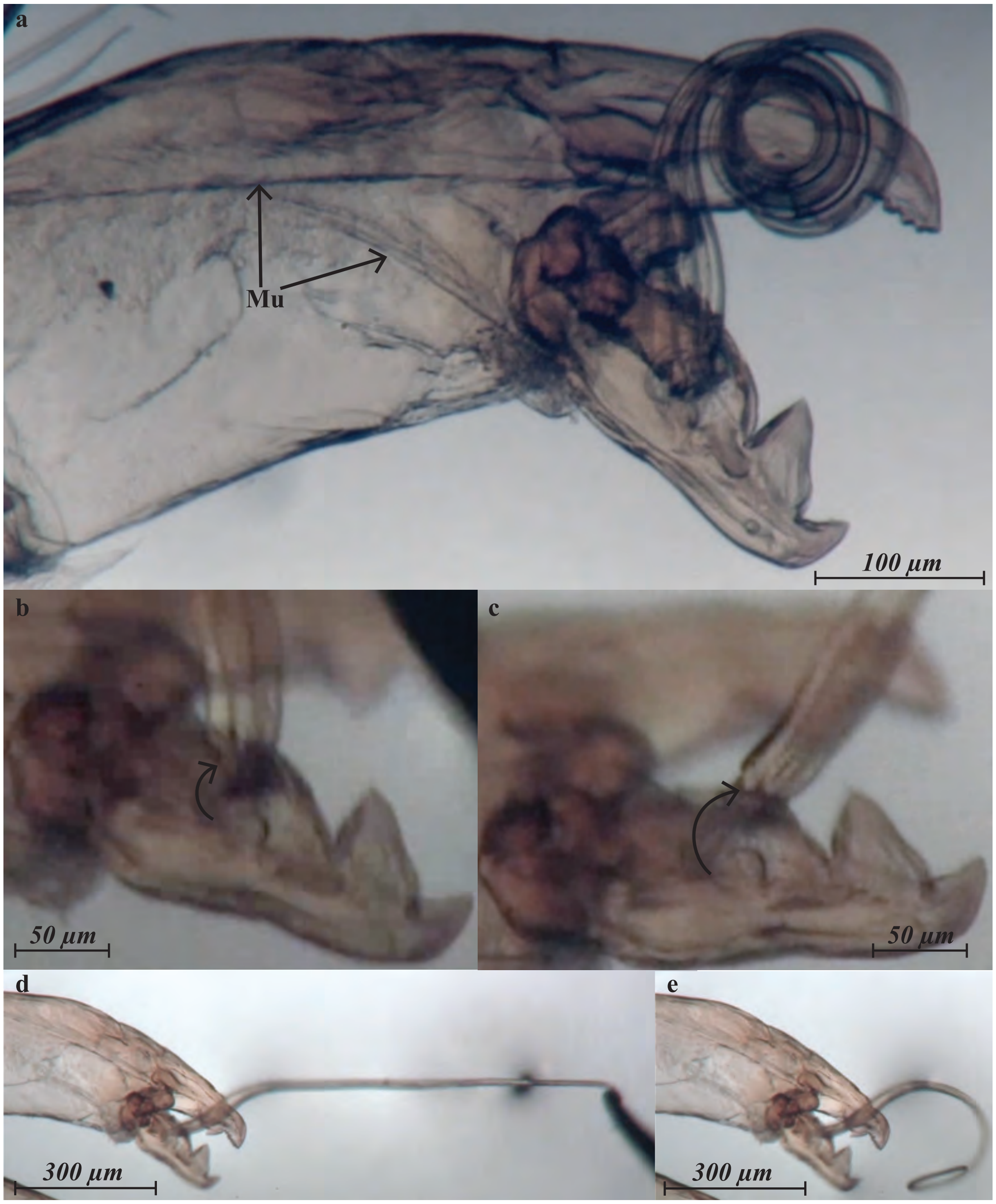

Fig 9. *Megalolaelaps colossus* male spermatodactyl under dissecting scope (a) detail of muscles (b) articulation retracted (c) articulation extended (d) spermatodactyl stretched (e) spermatodactyl going back to normal position. Mu, muscle.

Light Microscopy

Mites were cleared with lactic acid, dissected and mounted in Hoyer's medium on microscope slides and observed using differential interference contrast microscopy (DIC) using a Nikon Eclipse 90i (Melville, NY) microscope equipped with a motorized head with 10X, Plan Apo 20X, 40X, 40Xoil, and 100Xoil objectives and a PC controlled Ds-5M-U1 digital camera at the Acarology Laboratory of the Ohio State University (OSAL).

Scanning Electron Microscopy (SEM)

Mites were directly placed on aluminum studs on a disk-sample holder of a Hitachi TM3030Plus Tabletop SEM (Tarrytown, NY) equipped with a proprietary, highly sensitive low-vacuum secondary electron detector. The low-vacuum mode allowed us to image the mites without any coating or dehydration processing.

Low Temperature Scanning Electron Microscopy (LT-SEM) (ECMU)

Mites were observed through LT-SEM as described in Bolton et al. [20]. Briefly, mites preserved in ethanol were allowed to dry for a short time then secured to 15 mm x 30 mm copper plates using ultra smooth, round (12 mm diameter), carbon adhesive tabs (Electron Microscopy Sciences, Inc., Hatfield, PA, USA). The specimens were frozen conductively, in a Styrofoam box, by placing the plates on the surface of a pre-cooled (-196 °C) brass bar whose lower half was submerged in liquid nitrogen (LN2). After 20–30 s, the holders containing the frozen samples were transferred to a Quorum PP2000 cryo-prep chamber (Quorum Technologies, East Sussex, UK) attached to an S-4700 field emission scanning electron microscope (Hitachi High Technologies America, Inc., Dallas, TX, USA). The specimens were etched inside the cryo-transfer system to remove any surface contamination (condensed water vapor) by raising the temperature of the stage to -90°C for 10-15 min. Following etching, the temperature inside the chamber was lowered below -130°C, and the specimens were coated with a 10nm layer of platinum using a magnetron sputter head equipped with a platinum target. The specimens were transferred to a pre-cooled (-130 °C) cryo-stage in the SEM for observation. An accelerating voltage of 5kV was used to view the specimens. Images were captured using a 4pi Analysis System (Durham, NC).

CLSM

Images were obtained using three different confocal laser scanning microscopes systems, with mites mounted in different conditions as follows: at the Campus Microscopy and Imaging Facility (CMIF) at OSU we used an inverted Zeiss LSM880 microscope with Airyscan and one Olympus FV1000-Filter confocal system equipped with a upright Olympus BX61F/BX62; mites were on a microscope slides with Hoyer's medium or a concavity microscope slide with glycerin; at the ECMU images were obtained using an inverted Zeiss LSM710 system (Thornwood, NY) with mites in Hoyer's medium or placed in glycerin between two coverslips as described in Gulbronson et al. [38]. The LSM710 used 3 lasers simultaneously: a 405nm Diode laser, 488nm Argon laser, and a 561 DPSS laser, with a pin hole of 30 microns, and a filter set capturing emission between 410-483nm for blue; 495-553nm for green and 566-703nm for red to obtain 82–354 Z-stack images. The Olympus FV1000-Filter system used 4 lasers simultaneously: a 405nm Diode laser, 488nm Argon laser, a 543nm HeNe1, and a 633nm HeNe2, with a pin hole of 120 microns and a filter set capturing emission between

430-470nm for blue, 505-525nm for green, 560-660nm for yellow-orange, and 655-755nm for red to obtain 71–181 Z-stack images. The LSM880 used 1 or 2 lasers simultaneously: a 405nm Diode laser, and a 561 DPSS laser with a pin hole of 35 microns passing through a MBS 488/561/633 main dichroic beam splitter filter and a MBS 405 filter, with detection filters 459 and 578 Airyscan to obtain 483–503 Z-stack images. Complete parameters for individual images are included in Table 6 with their respective Morph Bank ID [39].

Image Enhancement

Z-stack images in Zeiss (.czi) or Olympus format (.oib) were opened and processed with the free software FIJI (Version 2.0.0; [40]), using the free-hand tool and the plugin 3D Scrip interactive animation [41] to isolate the region of interest (ROI), then the stack was saved in .tiff format. Tiff images were processed in two ways: 1) to convert Z-stack images into 3D models, FIJI was used to make the image binary, built the surface model and exported it in .stl format; then the .stl file was processed in Mesh Lab version 2020.06 [42] to remove unwanted regions, smooth surfaces, and to export the model to .u3d format to make it compatible with pdf. 2) using the open-source scientific visualization software Drishti [43]. In Drishti ImportTM, the tiff image was opened, and the histogram adjusted, after which it was saved in Drishti format (.nc). This image was opened in DrishtiTM for volume rendering. In this interface, opacity, density and gradient could be modified using the transfer and opacity tools to depict the ROI in high resolution. Then, when the rendered visualization was optimal, it was saved and exported as an .png image. Two dimensional images such as Maximum Intensity Projection (MIP) from stack, SEM, or LT-SEM or .pgn format from DrishtiTM, were modified in Photoshop Adobe Photoshop (Version 21.1.1; Adobe Systems Inc., San Jose, CA).

6 Acknowledgements

Thanks to Konstantin Thierbach and Genevieve Penzone for their valuable help with the imaging processing. Thanks to Andrew Ulsamer, Debra Creel and Joseph Mowery for helping with literature and loan of specimens. This project was partially funded by the fellowship ‘Doctorados Exterior’ 568-2012- C.C. 80048516 Colciencias, Colombia (OCH). Mention of trade names or commercial products in this publication is solely for the purpose of providing specific information and does not imply recommendation or endorsement by the U.S. Department of Agriculture. USDA is an equal opportunity provider and employer. Some images presented in this report were generated using the instruments and services at the Campus Microscopy and Imaging Facility. This facility is supported in part by grant P30 CA016058, National Cancer Institute, Bethesda, MD.

References

1. W. G. Eberhard, *Sexual Selection and Animal Genitalia.* Harvard University Press, jan 2013.

2. M. Ah-King, A. B. Barron, and M. E. Herberstein, “Genital Evolution: Why Are Females Still Understudied?,” *PLoS Biology*, vol. 12, no. 5, 2014.

3. N. S. Sloan and L. W. Simmons, “The evolution of female genitalia,” *Journal of Evolutionary Biology*, vol. 32, no. 9, pp. 882–899, 2019.

4. P. L. Brennan and R. O. Prum, “Mechanisms and evidence of genital coevolution: The roles of natural selection, mate choice, and sexual conflict,” *Cold Spring Harbor Perspectives in Biology*, vol. 7, no. 7, pp. 1–21, 2015.

5. P. L. Brennan, R. O. Prum, K. G. McCracken, M. D. Sorenson, R. E. Wilson, and T. R. Birkhead, “Coevolution of Male and Female Genital Morphology in Waterfowl,” *PLoS ONE*, vol. 2, p. e418, may 2007.

6. G. W. Krantz and D. E. Walter, *A Manual of Acarology.* Texas Tech University Press, third ed. ed., 2009.

7. A. Di Palma, O. Seeman, and G. Alberti, “Ultrastructure investigation of the secondary insemination system of the gamasid mite Hattena cometis Domrow (Acari: Anactinotrichida: Ameroseiidae),” *Journal of Morphology*, vol. 274, pp. 918–925, aug 2013.

8. D. E. Walter and H. C. Proctor, *Mites: Ecology, Evolution & Behaviour: Life at a Microscale.* Dordrecht: Springer, second ed. ed., 2013.

9. O. Cómbita-Heredia, E. J. Quintero-Gutiérrez, and H. Klompen, “Ontogeny of Megalolaelaps colossus sp. nov. (Acari: Megalolaelapidae), an enigmatic symbiont of dung beetles (Coleoptera: Scarabaeidae) in Colombia,” *Systematic and Applied Acarology*, vol. 23, no. 6, pp. 1102–1124, 2018.

10. G. Alberti and A. Di Palma, “Fine structure of the phytoseiid-type sperm access system (Acari, Gamasida, Phytoseiidae),” in *Acarid Phylogeny and Evolution: Adaptation in Mites and Ticks* (F. Bernini, R. Nannelli, G. Nuzzaci, and E. de Lillo, eds.), pp. 241–252, Dordrecht: Springer, Dordrecht, 2002.

11. G. Alberti, A. Di Palma, G. W. Krantz, and C. Błaszak, “First ultrastructural observations on a putative sperm access system in veigaiid females (Veigaiidae, Gamasida),” in *Trends in Acarology* (M. Sabelis and J. Bruin, eds.), pp. 59–64, Springer, Dordrecht, 2010.

12. A. Di Palma, G. J. de Moraes, B. S. Gerdeman, S. Huber, E. W. Kitajima, and G. Alberti, “Ultrastructural and functional adaptations of the female reproductive system in the family Heterozerconidae (Acari, Anactinotrichida, Gamasida, Heterozerconina) and implications for the systematic position of the group,” *Arthropod Structure and Development*, vol. 44, no. 6, pp. 639–655, 2015.

13. A. Di Palma, O. D. Seeman, and G. Alberti, “Complexity, adaptations and variations in the secondary insemination system of female Dermanyssina mites (Acari: Anactinothrichida: Gamasida): the case of Afrocypholaelaps africana,” *Experimental and Applied Acarology*, pp. 1–13, 2017.

14. A. Di Palma, G. Alberti, G. Nuzzaci, and G. W. Krantz, “Fine structure and functional morphology of the mouthparts of a maleVeigaia sp. (Gamasida: Veigaiidae) with remarks on the spermatodactyl and related sensory structures,” *Journal of Morphology*, vol. 267, pp. 208–220, feb 2006.

15. A. Di Palma, B. Gerdeman, and G. Alberti, “Fine structure and functional morphology of the spermatodactyl in males of Heterozerconidae (Gamasida),” *International Journal of Acarology*, vol. 34, pp. 359–366, 2008.

16. A. Di Palma, A. Wegener, and G. Alberti, “On the ultrastructure and functional morphology of the male chelicerae (gonopods) in Parasitina and Dermanyssina mites (Acari: Gamasida),” *Arthropod Structure and Development*, vol. 38, no. 4, pp. 329–338, 2009.

17. A. Di Palma, O. Seeman, and G. Alberti, “Ultrastructure of the male chelicerae of Hattena cometis Domrow (Acari: Gamasida: Ameroseiidae) functioning as gonopods,” *Journal of Morphology*, vol. 274, pp. 404–411, apr 2013.

18. C. Błaszak and R. Ehrnsberger, “Beschreibung des Typusmaterials von Veigaia paradoxa Willmann, 1951 (Acari, Gamasida: Veigaiaidae),” *Osnabrücker Naturwissenschaftliche Mitteilungen*, vol. 27, no. January 2001, pp. 89–97, 2001.

19. P. L. Brennan, “Studying Genital Coevolution to Understand Intromittent Organ Morphology,” in *Integrative and Comparative Biology*, vol. 56, pp. 669–681, Oxford University Press, oct 2016.

20. S. J. Bolton, H. Klompen, and G. R. Bauchan, “A new genus and species of Nematalycidae (Acari: Endeostigmata),” *Journal of Natural History*, vol. 48, no. 23-24, pp. 1359–1373, 2014.

21. S. D. Ramsey, R. Ochoa, G. Bauchan, C. Gulbronson, J. D. Mowery, A. Cohen, D. Lim, J. Joklik, J. M. Cicero, J. D. Ellis, D. Hawthorne, and D. VanEngelsdorp, “Varroa destructor feeds primarily on honey bee fat body tissue and not hemolymph,” *Proceedings of the National Academy of Sciences*, vol. 116, no. 5, pp. 1792–1801, 2019.

22. P. E. Chetverikov, “Confocal laser scanning microscopy technique for the study of internal genitalia and external morphology of eriophyoid mites (Acari: Eriophyoidea),” *Zootaxa*, vol. 3453, pp. 56–68, 2012.

23. F. Gorirossi-Bourdeau, “The gnathosoma of Megalolaelaps ornata (Acarina-Mesostigmata-Gamasides),” *The American Midland Naturalist*, vol. 55, no. 2, pp. 357–362, 1956.

24. J. P. Woodring and C. A. Galbraith, “The anatomy of the adult uropodid Fuscouropoda agitans (Arachnida; Acari), with comparative observations on other Acari,” *Journal of Morphology*, vol. 150, pp. 19–58, sep 1976.

25. B. D. Metscher, “Micro CT for comparative morphology: Simple staining methods allow high-contrast 3D imaging of diverse non-mineralized animal tissues,” *BMC Physiology*, vol. 9, no. 1, 2009.

26. P. E. Chetverikov, “Comparative confocal microscopy of internal genitalia of phytoptine mites (Eriophyoidea, Phytoptidae): New generic diagnoses reflecting host-plant associations,” *Experimental and Applied Acarology*, vol. 62, no. 2, pp. 129–160, 2014.

27. P. E. Chetverikov, “Distal oviduct and genital chamber of eriophyoids (Acariformes, Eriophyoidea): refined terminology and remarks on CLSM technique for studying musculature of mites,” *Experimental and Applied Acarology*, vol. 64, no. 4, pp. 407–428, 2014.

28. P. E. Chetverikov, “Confocal microscopy reveals uniform male reproductive anatomy in eriophyoid mites (Acariformes, Eriophyoidea) including spermatophore pump and paired vasa deferentia,” *Experimental and Applied Acarology*, vol. 66, no. 4, pp. 555–574, 2015.

29. P. E. Chetverikov, “New species and records of phytoptid mites (Acari: Eriophyoidea: Phytoptidae) on sedges (Cyperaceae) from the Russian Far East,” *Zootaxa*, vol. 4061, no. 4, pp. 367–380, 2016.

30. P. E. Chetverikov, F. Beaulieu, T. Cvrković, B. Vidović, and R. U. Petanović, "Oziella sibirica (Acari: Eriophyoidea: Phytoptidae), a new eriophyoid mite species described using confocal microscopy, COI barcoding and 3D surface reconstruction," *Zootaxa*, vol. 60, pp. 41–60, 2012.

31. P. E. Chetverikov, C. Craemer, A. E. Vishnyakov, and S. I. Sukhareva, "CLSM anatomy of internal genitalia of Mackiella reclinata n. sp. and systematic remarks on eriophyoid mites from the tribe Mackiellini Keifer, 1946 (Eriophyoidea: Phytoptidae)," *Zootaxa*, vol. 3860, no. 3, pp. 261–279, 2014.

32. P. E. Chetverikov, A. G. Desnitskiy, and D. Navia, "Confocal microscopy refines generic concept of a problematic taxon: Rediagnosis of the genus Neoprothrix and remarks on female anatomy of eriophyoids (Acari: Eriophyoidea)," *Zootaxa*, vol. 3919, no. 1, pp. 179–191, 2015.

33. P. E. Chetverikov and C. Craemer, "Gnathosomal interlocking apparatus and remarks on functional morphology of frontal lobes of eriophyoid mites (Acariformes, Eriophyoidea)," *Experimental and Applied Acarology*, vol. 66, no. 2, pp. 187–202, 2015.

34. P. L. Brennan, C. J. Clark, and R. O. Prum, "Explosive eversion and functional morphology of the duck penis supports sexual conflict in waterfowl genitalia," *Proceedings of the Royal Society B: Biological Sciences*, vol. 277, no. 1686, pp. 1309–1314, 2010.

35. O. Cómbita-Heredia, E. J. Quintero-Gutiérrez, N. Romero-García, and H. Klompen, "The biology of Megalolaelaps colossus (Acari: Dermanyssina)," *Experimental and Applied Acarology*, vol. 80, no. 2, pp. 167–181, 2020.

36. T. Tregenza, N. Wedell, and T. Chapman, "Introduction. Sexual conflict: A new paradigm?," *Philosophical Transactions of the Royal Society B: Biological Sciences*, vol. 361, no. 1466, pp. 229–234, 2006.

37. G. Arnqvist and L. Rowe, "Antagonistic coevolution between the sexes in a group of insects," *Nature*, vol. 415, pp. 787–789, feb 2002.

38. C. J. Gulbronson, J. Mowery, C. Pooley, R. Ochoa, S. Bolton, and G. Bauchan, "Three-Dimensional Printing of Agriculturally Important Mites Generated from Confocal Microscopy," *Microscopy and Microanalysis*, vol. 24, no. S1, pp. 1360–1361, 2018.

39. M. A. O'Leary and S. Kaufman, "MorphoBank: phylophenomics in the "cloud"," *Cladistics*, vol. 27, pp. 529–537, 2011.

40. J. Schindelin, I. Arganda-Carreras, E. Frise, V. Kaynig, M. Longair, T. Pietzsch, S. Preibisch, C. Rueden, S. Saalfeld, B. Schmid, J.-Y. Tinevez, D. J. White, V. Hartenstein, K. Eliceiri, P. Tomancak, and A. Cardona, "Fiji: an open-source platform for biological-image analysis," *Nature Methods*, vol. 9, pp. 676–682, jul 2012.

41. B. Schmid, P. Tripal, T. Fraaß, C. Kersten, B. Ruder, A. Grüneboom, J. Huisken, and R. Palmisano, "3Dscript: animating 3D/4D microscopy data using a natural-language-based syntax," *Nature Methods*, vol. 16, pp. 278–280, apr 2019.

42. P. Cignoni, M. Callieri, M. Corsini, M. Dellepiane, F. Ganovelli, and G. Ranzuglia, "MeshLab: An open-source mesh processing tool," *6th Eurographics Italian Chapter Conference 2008 - Proceedings*, no. January, pp. 129–136, 2008.

43. A. Limaye, "Drishti: a volume exploration and presentation tool," *Developments in X-Ray Tomography VIII*, vol. 8506, no. October 2012, p. 85060X, 2012.

Supporting information

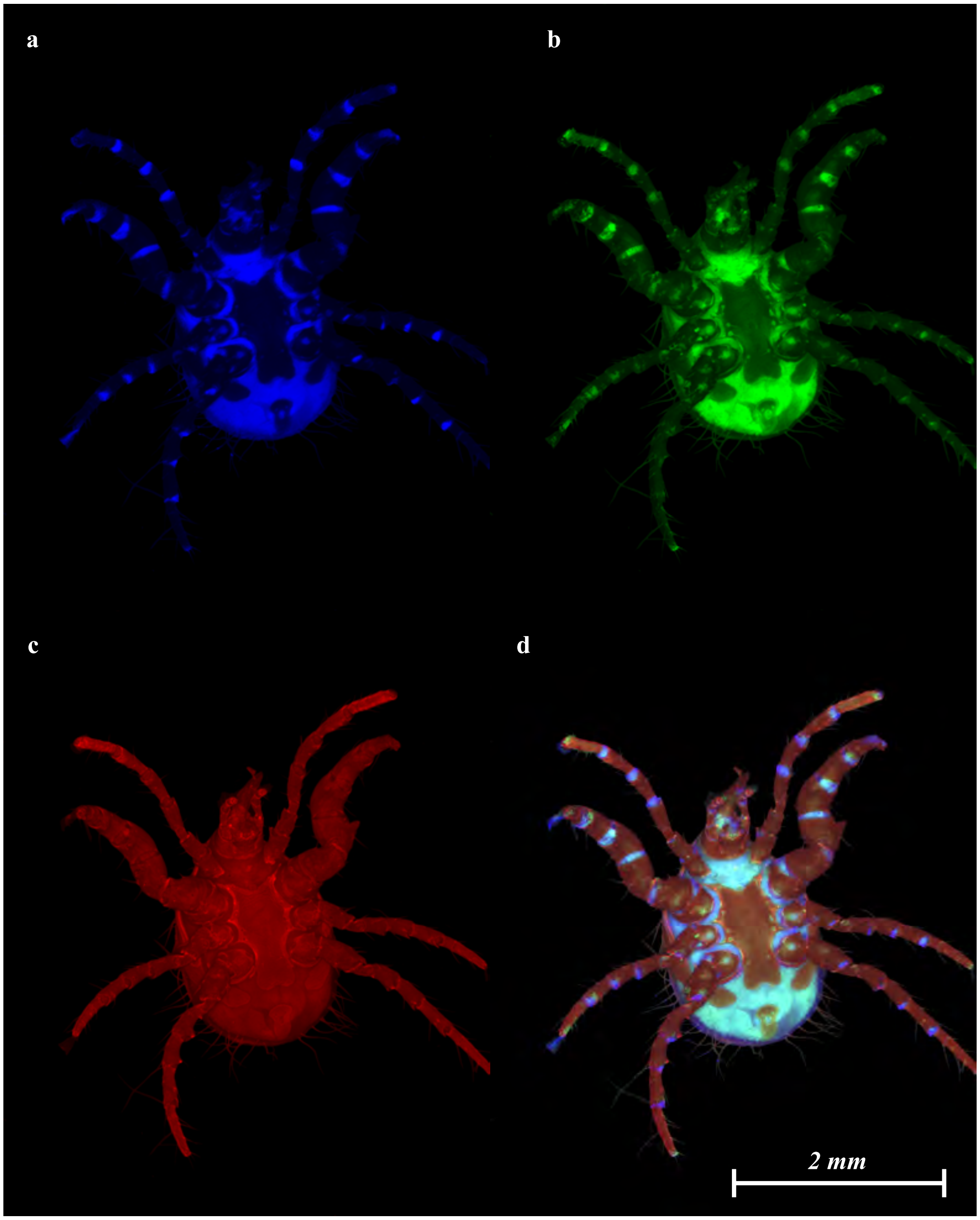

S1 Fig. *Megalolaelaps colossus* female ventral view. Fluorescence stereomicroscopy image: (a) blue filter (b) green filter (c) red filter (d) combined.

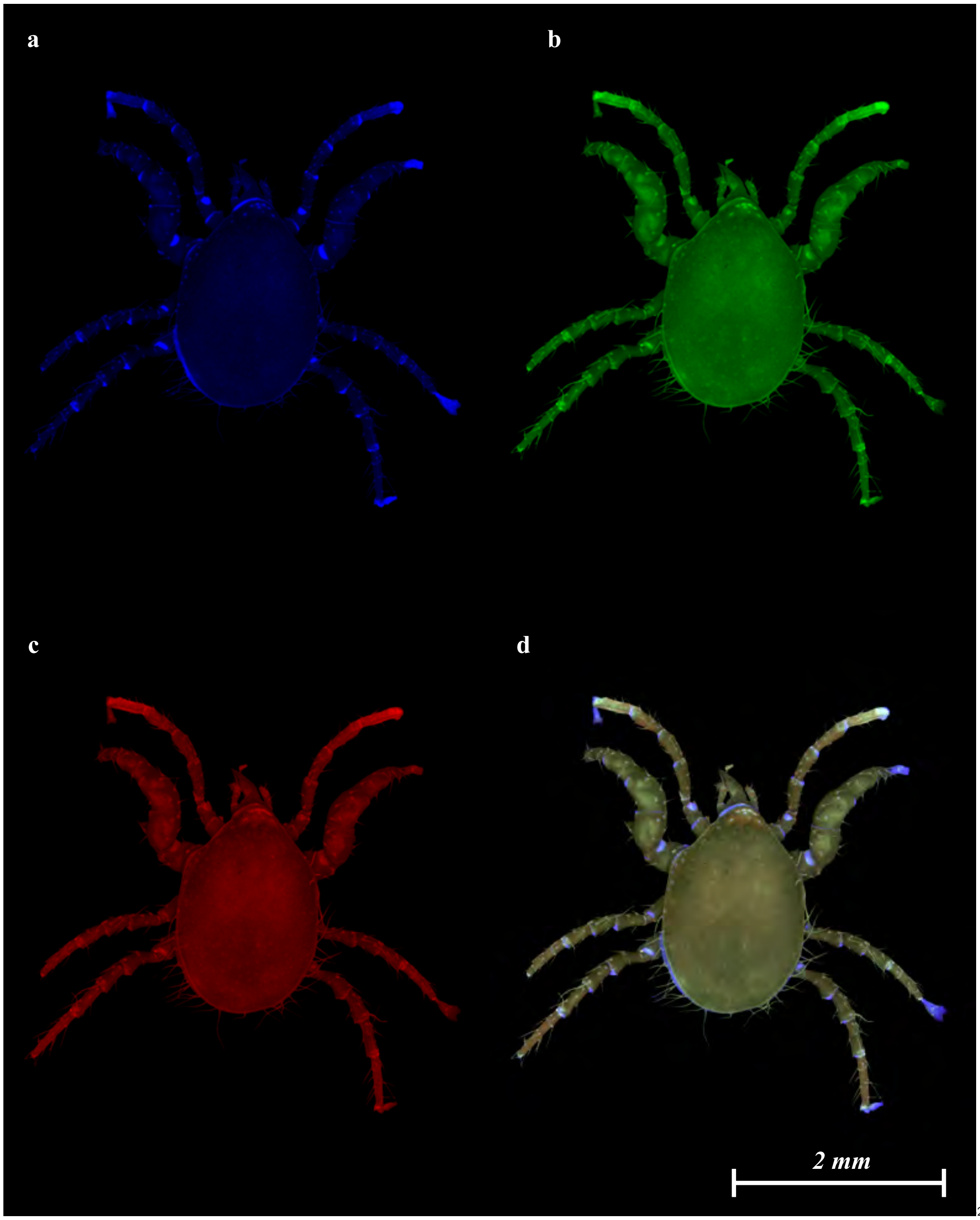

S2 Fig. ***Megalolaelaps colossus*** **male dorsal view**. Fluorescence stereomicroscopy image: (a) blue filter (b) green filter (c) red filter (d) combined.

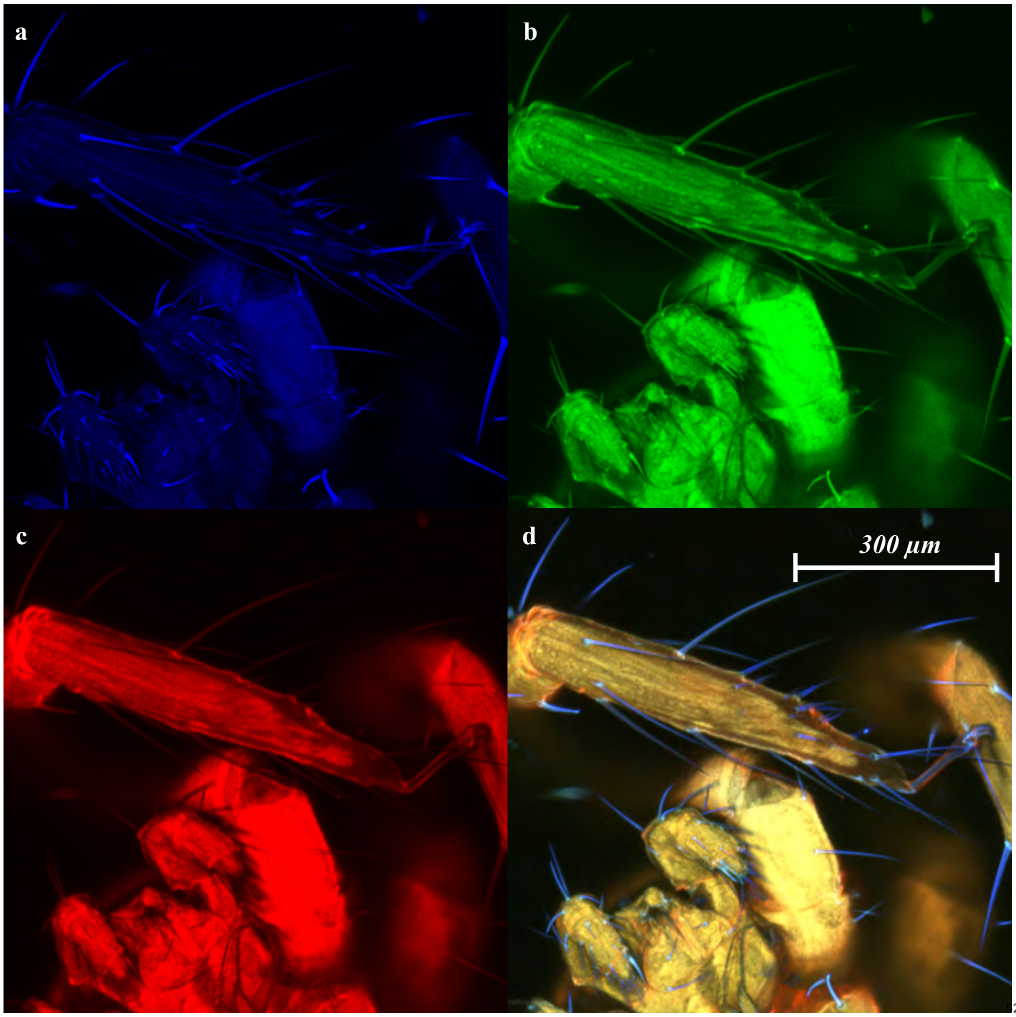

S3 Fig. ***Megalolaelaps colossus* larvae**. MIP images of tarsus I with CLSM: (a) blue channel (b) green channel (c) red channel (d) combined.

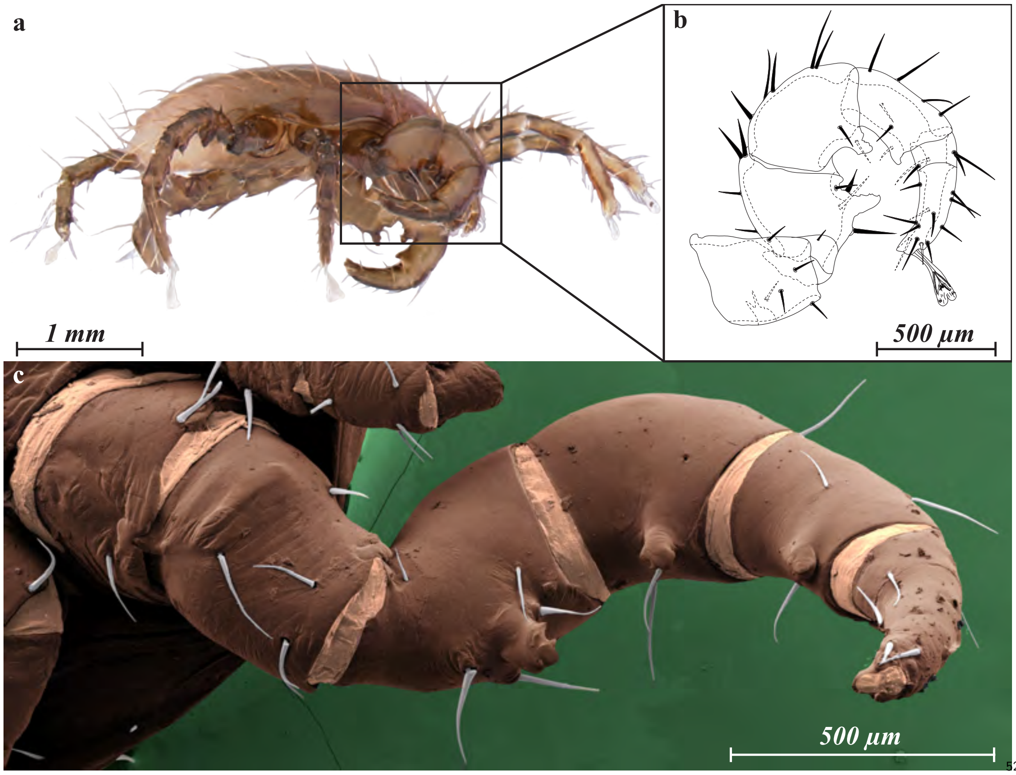

S4 Fig. ***Megalolaelaps colossus*** **male**. Leg II: (a) colorized LT-SEM of lateral view (b) drawing the detail of leg II (c) colorized LT-SEM image.

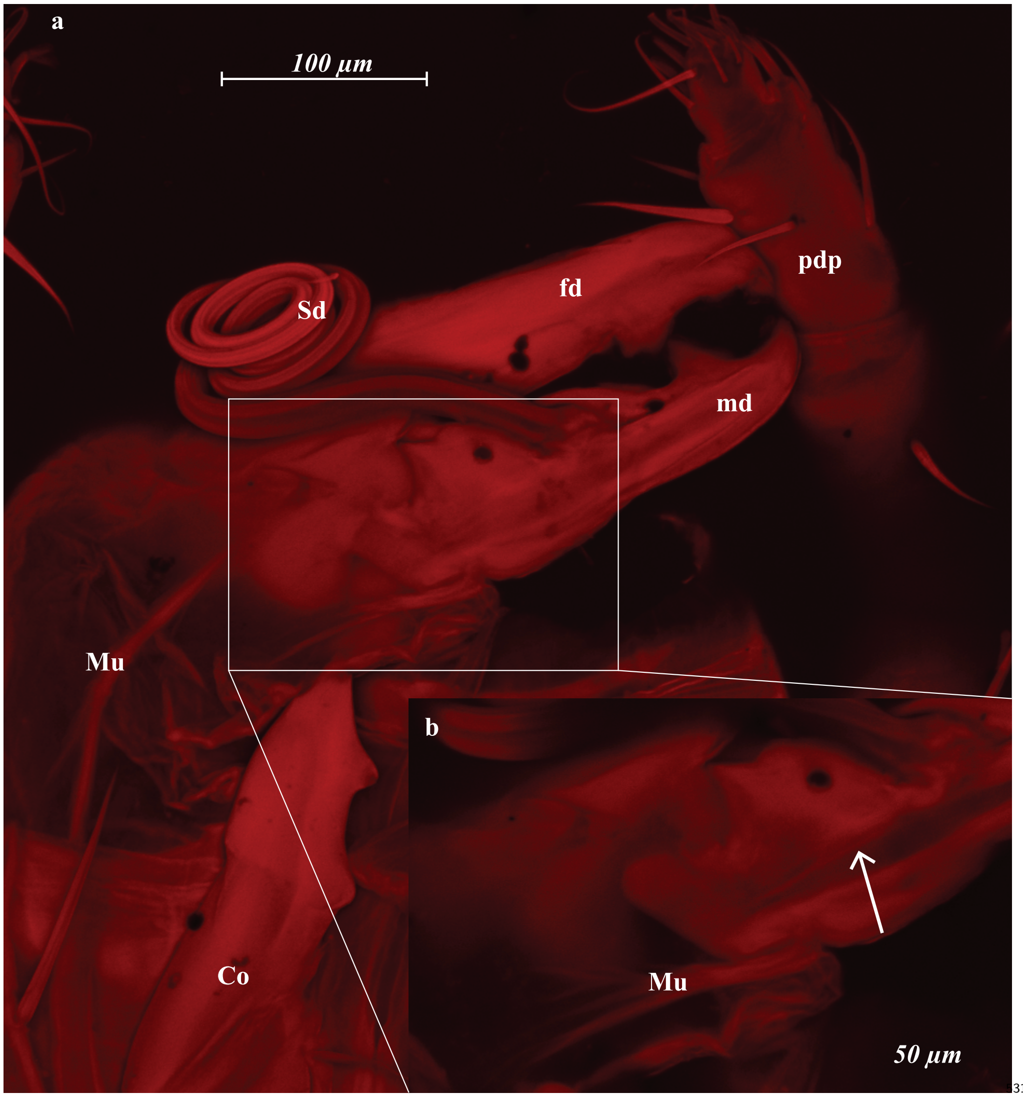

S5 Fig. ***Megalolaelaps enceladus* male**. Spermatodactyl and muscle fibers (arrow). Co, corniculi; fd, fixed digit; md, movable digit; Mu, muscle; pdp, pedipalp; Sd, spermatodactyl.

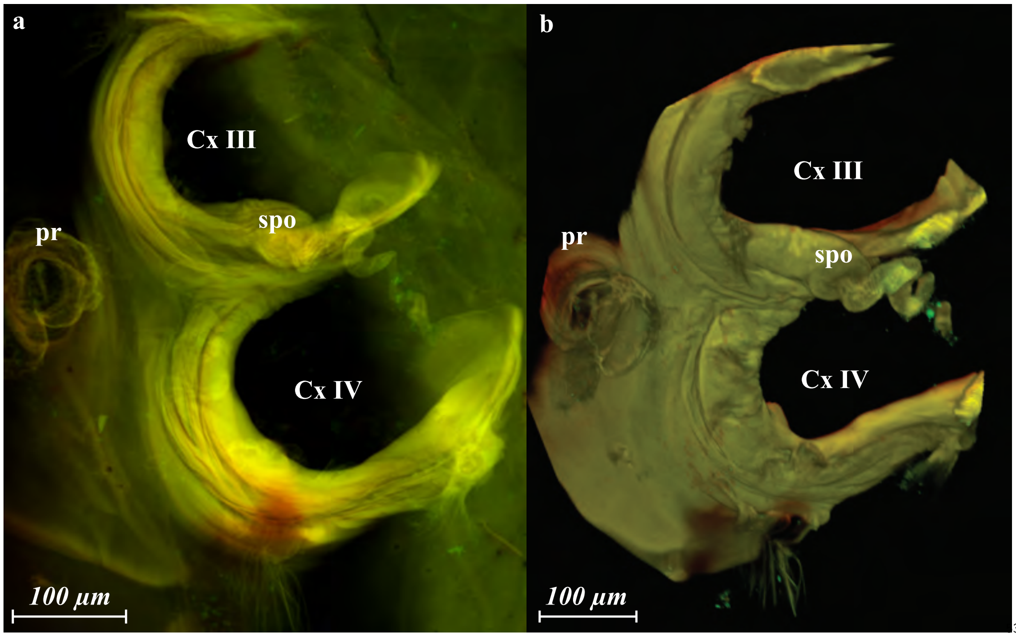

S6 Fig. ***Megalolaelaps enceladus* female**. Slide prepared in 1930 with Hoyer's medium. Region of coxae III–IV from dorsum to venter (a) MIP image from CLSM (b) rendered model in FIJI. Cx I–II, coxa I–II; pr, peritreme; spo, spiral organ.

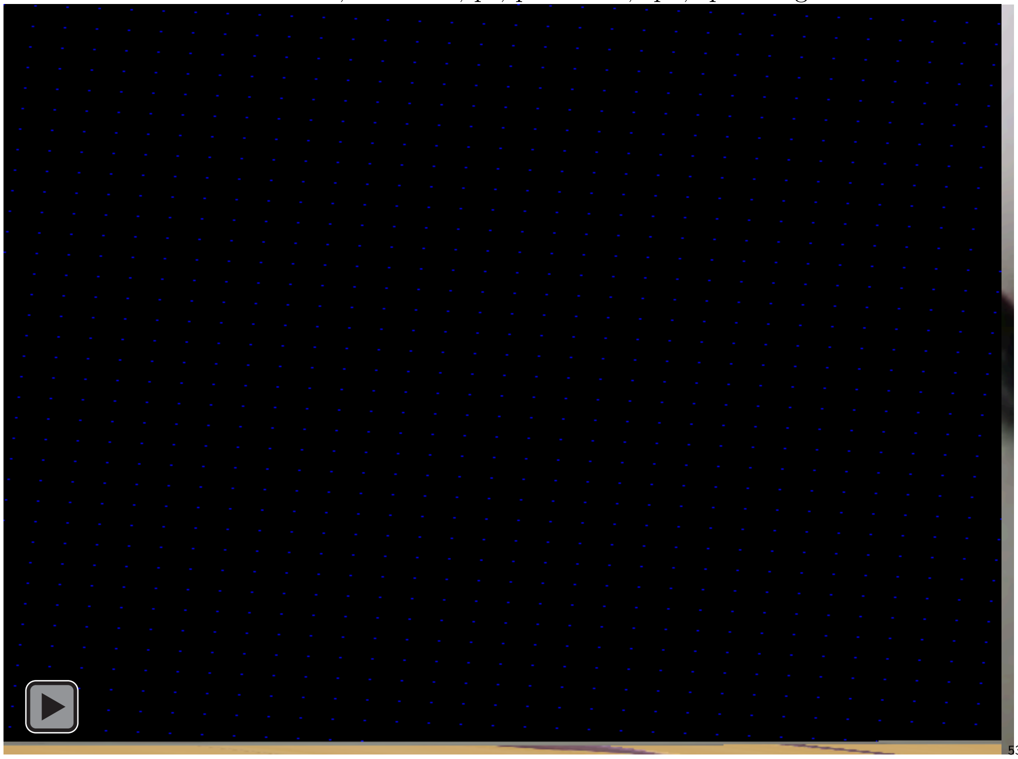

S1 Video. ***Megalolaelaps enceladus* male**. video: spermatodactyl under dissecting scope with detail of muscles, articulation and its motility. Demonstrates how the spermatodactyl can be "unfolded" and how it returns back to normal position by elasticity.

Fig. #	Specimen	Instar	Structure	Medium	Facility	Technique	Microscope	Objective	Lasers	Pin hole (µm)	Excitation Filter	Detection parameters	Size	Z Stack	Voucher number	Morphobank ID
**1**	*M. colossus*	Adult male	Chelicerae and spermatodactyl	Hoyer's	CMIF	CLSM	Zeiss LSM880	Plan Apochromat 20x/0.8 M27	561nm DPSS	35	MBS 488/561/633, MBS -405	578 Airyscan	3.5GB	483	OSAL 0099605	M687595
**2a**	*M. colossus*	Adult male	Whole body dorsal view	None	ECMU	SEM	TM3030	N/A	N/A	N/A	N/A	N/A	N/A	N/A	N/A	M692293
**2b**	*M. colossus*	Adult male	Chelicerae and spermatodactyl	None	ECMU	SEM	TM3030	N/A	N/A	N/A	N/A	N/A	N/A	N/A	N/A	M692294
**2c**	*M. colossus*	Adult male	Chelicerae and spermatodactyl	Glycerin	CMIF	CLSM	Olympus FV1000-Filter	UPLFLN 20X NA:0.50	405nm Diode laser, 488nm Argon laser, 543nm HeNe1, 633nm HeNe2	120	DM405/488/543/635	430-470nm for blue, 505-525nm for green, 560-660nm for yellow-orange, and BA655-755 for red	149MB	122	N/A	M692295
**S1a-d**	*M. colossus*	Adult male	Whole body ventral view	None	ECMU	FSt	Zeiss AxioZoom	1x 0.25NA PlanNeoFluor	N/A	N/A	N/A	N/A	N/A	N/A	N/A	M692281
**S2 a-d**	*M. colossus*	Adult male	Whole body dorsal view	None	ECMU	FSt	Zeiss AxioZoom	1x 0.25NA PlanNeoFluor	N/A	N/A	N/A	N/A	N/A	N/A	N/A	M692282
**S3 a-d**	*M. colossus*	Larvae	Tarsus 1	Glycerin	ECMU	CLSM	Zeiss LSM710	Fluar 10x/0.50	405nm Diode, 488nm Argon, 561nm DPSS	35	MBS 488/561, MBS 405	410-483nm blue; 495 - 553nm green, 566-703nm red	246MB	210	N/A	M692288
**3a**	*M. colossus*	Adult male	Chelicerae and spermatodactyl	None	ECMU	LT-SEM	S-4700 LTSEM	N/A	N/A	N/A	N/A	N/A	N/A	N/A	N/A	M692294
**3b**	*M. colossus*	Adult male	Chelicerae and spermatodactyl	Hoyer's	CMIF	CLSM	Olympus FV1000-Filter	UPLFLN 40X O NA:1.30	405nm Diode laser, 488nm Argon laser, 543nm HeNe1	75	DM405/488/543	430-470nm for blue, 505-525nm green, 560-660nm yellow-orange	663MB	181	N/A	M692294
**4a-e**	*M. colossus*	Adult male	Spermatodactyl	Hoyer's	CMIF	CLSM	Olympus FV1000-Filter	UPLFLN 40X O NA:1.30	405nm Diode laser, 488nm Argon laser, 543nm HeNe1	75	DM405/488/543	430-470nm for blue, 505-525nm green, 560-660nm yellow-orange	663MB	181	N/A	M692295
**5**	*M. colossus*	Adult female	Coxae II-IV ventrally	None	ECMU	SEM	TM3030	N/A	N/A	N/A	N/A	N/A	N/A	N/A	N/A	M692296
**6a**	*M. colossus*	Adult female	Detail of the spiral organ	Hoyer's	OSAL	DIC	Nikon Eclipse 90i	Plan Apo 40X/0.75	N/A	N/A	N/A	N/A	N/A	N/A	N/A	M692297
**6b**	*M. colossus*	Adult female	Detail of the spiral organ	Glycerin	OSAL	DIC	Nikon Eclipse 90i	Plan Apo 40X/0.75	N/A	N/A	N/A	N/A	N/A	N/A	N/A	M692297
**6c**	*M. colossus*	Adult female	Detail of the spiral organ	Glycerin	ECMU	CLSM	Zeiss LSM710	Fluar 10x/0.50	405nm Diode, 488nm Argon, 561nm DPSS	25	MBS 488/561, MBS 405	410-483nm blue; 495 - 553nm green, 566-703nm red	2.1GB	354	N/A	M692297
**6d**	*M. colossus*	Adult female	Detail of the spiral organ	Hoyer's	CMIF	CLSM	Zeiss LSM880	Plan-Neofluar 40x/1.30 Oil DIC M27	405nm Diode, 561nm DPSS	35	MBS 488/561/633, MBS -405	459/578 Airyscan	6.3GB	503	OSAL 0099606	M692297
**7a**	*M. colossus*	Adult male, female	Whole body	None	OSUC	St	Leica Z16	1X	N/A	N/A	N/A	N/A	N/A	N/A	N/A	M692315
**7b**	*M. colossus*	Adult male, female	Whole body lateral view	N/A	ECMU	LT-SEM	S-4700 LTSEM	LTSEM and Illustrator drawing	N/A	N/A	N/A	N/A	N/A	N/A	N/A	M692315
**7c**	*M. colossus*	Adult male, female	Detail of the spiral organ	Hoyer's	CMIF	CLSM	Zeiss LSM880	Plan-Neofluar 40x/1.30 Oil DIC M27	405nm Diode, 561nm DPSS	35	MBS 488/561/633, MBS -405	459/578 Airyscan	6.3GB	503	OSAL 0099606	M692311
**7d**	*M. colossus*	Adult male	Chelicerae and spermatodactyl	Glycerin	CMIF	CLSM	Olympus FV1000-Filter	UPLFLN 20X NA:0.50	405nm Diode laser, 488nm Argon laser, 543nm HeNe1, 633nm HeNe2	120	DM405/488/543/635	430-470nm blue, 505-525nm for green, 560-660nm yellow-orange, and BA655-755 red	149MB	122	OSAL 0099605	M692314
**S4a**	*M. colossus*	Adult male	Whole body lateral view	None	OSUC	St	Leica Z16	1X	N/A	N/A	N/A	N/A	N/A	N/A	N/A	M692298
**S4b**	*M. colossus*	Adult male	Leg II	None	OSAL	DIC	Illustration based on DIC	N/A	N/A	N/A	N/A	N/A	N/A	N/A	N/A	M692299
**S4c**	*M. colossus*	Adult male	Leg II ventral view	None	ECMU	LT-SEM	S-4700 LTSEM	N/A	N/A	N/A	N/A	N/A	N/A	N/A	N/A	M692300
**8**	*M. colossus*	Adult male	Coxae II-IV internally	Glycerin	CMIF	CLSM	Olympus FV1000-Filter	UPLSAPO 20X O NA:0.85	405nm Diode laser, 488nm Argon laser,543nm HeNe1, 633nm HeNe2	80	DM405/488/543/635	430-470nm for blue, 505-525nm green, 560-660nm yellow-orange, and BA655-755 red	440MB	55	N/A	M692299
**9a-e**	*M. colossus*	Adult male	Internal muscles of the chelicera	None	OSUC	St	Leica Z16	1X	N/A	N/A	N/A	N/A	N/A	N/A	N/A	M692300
**S5a-b**	*M. enceladus*	Adult male	Chelicera and spermatodactyl	Hoyer's	ECMU	CLSM	Zeiss LSM710	Fluar 10x/0.50	405nm Diode, 488nm Argon, 561nm DPSS	25	405nm Diode laser, 488nm Argon laser,543nm HeNe1, 633nm HeNe2	410-483nm blue; 495 - 553nm green, 566-703nm red	246 MB	82	N/A	M692301
**S6a-b**	*M. enceladus*	Adult female	Coxae II-III internally	Hoyer's	CMIF	CLSM	Olympus FV1000-Filter	UPLSAPO 20X O NA:0.85	405nm Diode laser, 488nm Argon laser,543nm HeNe1, 633nm HeNe2	80	DM405/488/543/635	430-470nm blue, 505-525nm green, 560-660nm yellow-orange, and BA655-755 red	284MB	71	NHM120	M692302

S1 Table. Individual parameters for the images.
